# SpectralTCN: A multi-resolution wavelet gating network for vibration-based structural damage detection

**DOI:** 10.1371/journal.pone.0358224

**Published:** 2026-09-21

**Authors:** Quan Pham Hong, Trung Vu Manh, Bich Nguyen Thach, Le Nguyen Dan, Hoa Tran Ngoc

**Affiliations:** 1 Faculty of Civil Engineering, University of Transport Technology, Ha Noi, Viet Nam; 2 Application of Artificial Intelligence for Structural Health Monitoring (AI-SHM) Research Group (R2026-UTC-01), Faculty of Civil and Environmental Engineering, University of Transport and Communications, Hanoi, Vietnam; National Changhua University of Education, TAIWAN

## Abstract

Vibration-based structural health monitoring (SHM) detects damage by identifying subtle changes in a structure’s dynamic response. Deep learning models have shown strong potential for automating this classification task. However, most existing architectures share a key limitation: they either process signals purely in the time domain, or apply a global frequency-domain transform that discards information about when each frequency component occurs. This is a serious drawback, because structural damage typically manifests as short, localised events confined to specific frequency bands. This paper proposes SpectralTCN, a temporal convolutional network augmented with a Wavelet Gating Module (WGM) that performs learnable, data-dependent multi-resolution filtering within each convolutional block. The WGM decomposes intermediate features via the Discrete Wavelet Transform (DWT) into physically interpretable sub-bands corresponding to distinct structural vibration modes, applies input-adaptive sigmoid gates independently at each decomposition level, and reconstructs the filtered signal via the Inverse DWT (IDWT) with a learnable residual connection initialised to zero. Unlike FFT-based approaches, the DWT simultaneously preserves both time and frequency information, enabling the network to detect both the timing and the spectral location of damage-induced anomalies. Combined with Generalized Mean (GeM) pooling and large-kernel causal depthwise convolutions, SpectralTCN is evaluated on two benchmark datasets: the Z24 Bridge benchmark and a finite element model (FEM)-derived dataset of the My Thuan cable-stayed bridge. Experiments against 10 baseline models and 4 ablation variants, evaluated via stratified 5-fold cross-validation, demonstrate the effectiveness and generalisation capability of the proposed approach: SpectralTCN attains the highest mean accuracy on both benchmarks (92.2% on Z24 and 92.1% on My Thuan) and outperforms the strongest baseline in the 5-fold cross-validation protocol. In addition, the proposed architecture operates on short, streaming acceleration windows with a purely convolutional backbone of moderate computational cost, making SpectralTCN suitable for **near-real-time, online damage detection** in continuous bridge monitoring.

## Introduction

Structural health monitoring (SHM) of civil infrastructure aims to detect and locate damage by continuously or periodically measuring structural responses [1]. This is especially important for maintaining the safety of large bridges. Among the available sensing modalities, vibration-based monitoring using accelerometers is the most widely adopted. This is because shifts in a structure’s dynamic characteristics (natural frequencies, damping ratios, and mode shapes) are direct indicators of damage [2]. This premise is further supported by experimental studies of structural systems under dynamic excitation. For instance, research on the response of group piles to vertical vibration during earthquake loading [3] confirms that dynamic-response characteristics are sensitive indicators of a structure’s state. Traditional SHM relies on physics-based modal analysis and visual inspection, both of which require substantial domain expertise and are sensitive to environmental confounders such as temperature variation and traffic loading that can mask damage signatures; these difficulties have motivated data-driven and metaheuristic-assisted identification schemes [4].

Data-driven approaches based on deep learning have emerged as a compelling alternative, enabling automated feature extraction directly from raw vibration signals without hand-crafted features. One-dimensional convolutional neural networks (1DCNNs) [5], long short-term memory (LSTM) networks [6], and Transformer-based models [7] have demonstrated strong performance on SHM classification tasks. More recently, Temporal Convolutional Networks (TCNs) [8] have gained traction in time-series modelling because their dilated causal convolutions and residual connections offer high computational efficiency and a large effective receptive field. ModernTCN [9] further advances this design with ConvNeXt-style blocks and large-kernel depthwise convolutions. However, a major limitation of these architectures is that they operate solely in the time domain, missing out on the critical frequency features of structural dynamics.

To address this limitation, several studies have sought to incorporate spectral information through Fourier-based processing. FNet [10], GFNet [11], and FreTS [12] demonstrated the utility of global frequency-domain operations for language and time-series tasks. However, the discrete Fourier transform (DFT) is inherently a global operation: by mapping the entire signal to the frequency domain, it completely discards temporal localisation.

The DWT addresses this limitation by providing simultaneous time-frequency resolution. It decomposes a signal into sub-bands that are localised in both time and frequency, with each decomposition level corresponding directly to a specific octave frequency band. While wavelet analysis has an established history as a preprocessing tool in SHM [13], its integration into deep learning models as a learnable, input-adaptive component embedded within network blocks remains largely unexplored.

This paper proposes SpectralTCN, a temporal convolutional network that integrates explicit multi-resolution wavelet processing through a novel Wavelet Gating Module (WGM). Building on the ModernTCN backbone, the WGM is inserted between each depthwise convolution and feed-forward layer. First, it decomposes intermediate feature maps via the DWT into sub-bands that align with structural vibration mode frequency bands. Subsequently, learnable input-adaptive sigmoid gates are applied independently at each decomposition level before the filtered signal is reconstructed via the inverse DWT within a zero-initialised residual connection. This design enables the model to selectively amplify or suppress specific frequency bands in a data-dependent manner without sacrificing temporal localisation. SpectralTCN further incorporates Generalized Mean (GeM) pooling [14] in the classification head to provide learnable temporal aggregation that adapts to localised damage signatures.

The model is evaluated on two datasets: the Z24 Bridge benchmark comprising 17 structural states recorded by 27 accelerometers [15], and a finite element model (FEM)-derived dataset of the My Thuan cable-stayed bridge in Vietnam simulating 10 structural damage scenarios. This hybrid validation strategy assesses the model under realistic, noisy operational conditions while also allowing a safe, controlled evaluation of its sensitivity to dense and progressive damage scenarios that cannot be executed on active bridges.

The main contributions of this work are:

1. A novel WGM that performs learnable, data-dependent multi-resolution filtering via the DWT within the convolutional blocks. Unlike FFT-based approaches, the WGM preserves simultaneous time-frequency resolution, with each decomposition level physically corresponding to a specific octave frequency band of structural vibration modes.
2. Integration of GeM pooling into the classification head, providing a learnable temporal aggregation mechanism that adapts to localised damage features.
3. An experimental evaluation on two structural datasets (the Z24 Bridge benchmark and the My Thuan bridge FEM dataset) using stratified 5-fold cross-validation. The proposed architecture is compared against 10 baseline models spanning convolutional, recurrent, Transformer, MLP, frequency-domain and state-space families, and analysed alongside 4 ablation variants, including a controlled DWT-versus-FFT comparison inside an identical backbone.

The remainder of this paper is organised as follows. The Related work section reviews existing literature. The Methodology section presents the SpectralTCN architecture. The Experiments section describes the experimental setup and results. The Conclusions section summarises the key findings.

## Related work

### Deep learning for structural health monitoring

The application of deep learning to vibration-based SHM has expanded substantially over the past decade. Early approaches employed one-dimensional convolutional neural networks (1DCNNs) applied directly to raw acceleration signals, circumventing the need for manual feature extraction and demonstrating competitive performance on damage classification tasks [5]. Simultaneously, LSTM networks were applied to model the temporal dependencies in structural vibration signals, with demonstrated capability for detecting gradual stiffness degradation and crack propagation [6]. Hybrid architectures that combine these two mechanisms have gained traction for bridge damage detection [16]: CNN-BiGRU models [17] and BiLSTM-1DCNN networks [18,19] leverage convolutional feature extraction alongside bidirectional recurrent context, demonstrating improved classification accuracy on steel truss bridge benchmarks. An effective hybrid framework combines symbolic aggregate approximation with a 1DCNN-BiGRU backbone to detect stiffness degradation in the Chuong Duong steel truss bridge under simulated damage scenarios [20]. Multi-scale architectures such as InceptionTime [21], which applies parallel convolutions of varying kernel sizes, further improved the capture of temporal patterns at multiple resolutions. More recently, Transformer-based models [7] have been adopted for SHM, exploiting self-attention to model global dependencies across sensor channels and time steps simultaneously. Data-driven frameworks for bridge structures have further combined discrete structural modelling with FFT-based feature extraction to enable automated SHM from measured acceleration responses [22]. In addition, deep ensemble strategies have shown promise for improving damage classification robustness in the presence of noise [23]. Complementary to the classification model itself, a parallel line of research addresses the data-acquisition stage of SHM, where optimal sensor layout design aims to maximise the observability of damage-sensitive responses [24]. Deep learning has likewise been extended beyond fixed accelerometer networks to mobile inspection platforms; for example, a two-stage CNN combined with lighthouse-based localisation enables UAV-based monitoring in GNSS-denied environments [25].

A distinct body of work incorporates spectral features as a preprocessing step. Power spectral densities, spectrograms, and wavelet scalograms computed offline have been supplied to standard CNN or multi-layer perceptron (MLP) classifiers [13,26]; however, such pipelines decouple spectral analysis from classification and require domain expertise to select appropriate transform parameters. SpectralTCN instead embeds learnable, data-dependent multi-resolution processing directly inside the convolutional blocks.

### Temporal convolutional networks

The TCN architecture [8] established dilated causal convolutions with residual connections as an effective alternative to recurrent models for sequence modelling, offering superior computational parallelism and a controllable receptive field. ModernTCN [9] extended this design with ConvNeXt-style [27] principles: large-kernel depthwise convolutions, layer normalisation, an inverted-bottleneck feed-forward network, and a hierarchical multi-stage structure, achieving state-of-the-art results on diverse time-series benchmarks. SpectralTCN builds directly on the ModernTCN backbone, augmenting each block with the WGM to introduce explicit multi-resolution frequency processing while preserving the efficiency and long-range modelling capability of large-kernel depthwise convolutions.

### Frequency-domain and wavelet processing in neural networks

The integration of frequency-domain operations into neural network architectures has attracted growing interest. FNet [10] demonstrated that replacing self-attention with Fourier mixing achieves competitive natural language processing (NLP) performance at substantially lower cost. GFNet [11] introduced globally learnable Fourier-domain filters applied to spatial feature maps for image classification. FreTS [12] proposed frequency-domain MLP blocks for multivariate time-series forecasting. State-space models (SSMs) such as MambaSL [28] represent a complementary direction, dynamically modulating state transitions conditioned on the input to achieve linear time complexity with strong long-range modelling capability. Despite this breadth, all FFT-based methods share a fundamental limitation: the DFT collapses the temporal axis entirely, providing global spectral estimates but discarding temporal localisation. SSMs implicitly capture certain spectral properties through their continuous-time parameterisation but do not offer explicit, physically interpretable decomposition into frequency sub-bands.

The DWT addresses this deficiency by providing simultaneous time-frequency resolution through a multi-resolution sub-band decomposition. Wavelet analysis has long served as the primary tool for time-frequency characterisation of structural vibration signals [13,29,30], with lower decomposition levels capturing high-frequency transients and higher levels capturing the lower modal frequency content. Despite this suitability, prior SHM studies treat wavelet transforms as fixed preprocessing operations [13]; the WGM proposed here closes this gap by combining input-adaptive gating with the physically interpretable decompositions that FFT-based methods cannot offer.

### Temporal aggregation in classification heads

The choice of temporal aggregation strategy in the classification head affects performance when discriminative features are unevenly distributed across time. Standard global average pooling treats all time steps equally and may dilute localised damage signatures, whereas max pooling is sensitive to noise. GeM pooling [14], originally introduced for image retrieval, interpolates between average and max pooling via a learnable exponent p optimised end-to-end, adapting the aggregation strategy to the statistical structure of the features.

## Methodology

This section presents the proposed SpectralTCN architecture for vibration-based structural damage classification. We first describe the overall model pipeline, then detail each core component: the ModernTCN Block with the proposed WGM, and the Classification Head with Generalized Mean Pooling.

### Overall model pipeline

**Fig 1:**
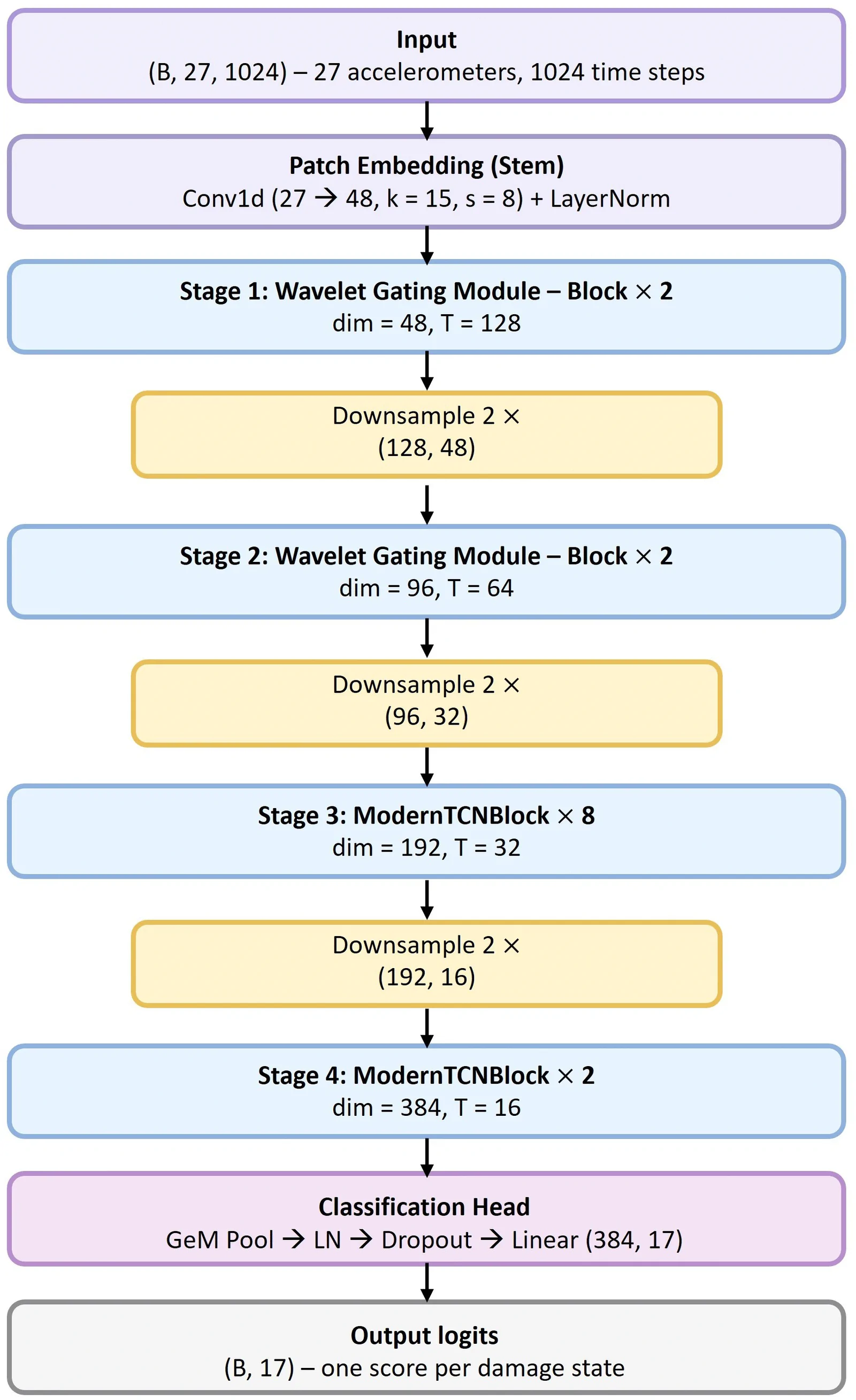
Overall SpectralTCN architecture.

SpectralTCN follows a hierarchical, multi-stage design inspired by ModernTCN [9] and ConvNeXt [27]. Given a raw multi-channel vibration signal $X_{\text{in}}\in R^{B\times C_{\text{in}}\times T}$, the forward pass proceeds as:


$$\begin{array}{c} \hat{y}=Head\left({Stage}_{S}\circ {Down}_{S-\text{1}}\circ \cdots \circ {Down}_{\text{1}}\circ {Stage}_{\text{1}}\circ Stem(X_{in})\right) \end{array}$$ (1)

where Stem is a patch embedding layer, each ${Stage}_{s}$ consists of $N_{s}$ stacked ModernTCN blocks operating at channel dimension $d_{s}$, ${Down}_{s}$ halves the temporal resolution while doubling the channel width, and Head maps the final feature map to class logits. The full pipeline is illustrated in Fig 1, and all hyperparameters are described in the Experiments section.

The stem converts raw sensor signals into patch-level token embeddings through a strided causal convolution followed by layer normalisation:


$$\begin{array}{c} Z^{\left(\text{0}\right)}=LN\left({Conv\text{1}d}_{k_{p},\;s_{p}}\left(CausalPad(X_{in})\right)\right)\in R^{B\times d_{\text{1}}\times T^{\prime }}, \end{array}$$ (2)

where $k_{p}$ = 15 is the stem kernel size, $s_{p}$ = 8 is the patch stride, causal padding prepends $k_{p}-s_{p}$ = 7 zeros to the left, and $T^{\prime }=T/s_{p}$ = 128. This reduces the temporal resolution by a factor of 8, substantially lowering computation in subsequent stages while preserving local temporal structure.

### ModernTCN block

**Fig 2:**
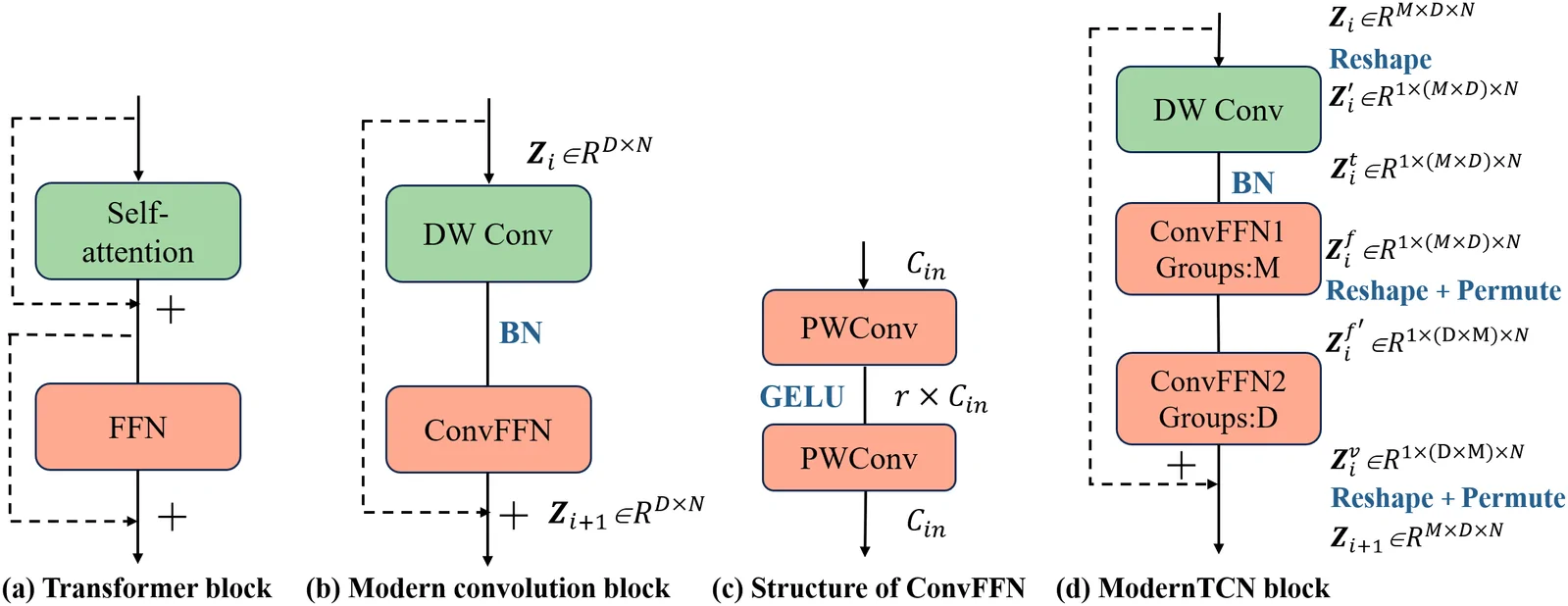
Architecture of the original ModernTCN block.

Each stage consists of $N_{s}$ identical ModernTCN blocks, whose base design is illustrated in Fig 2.

**Fig 3:**
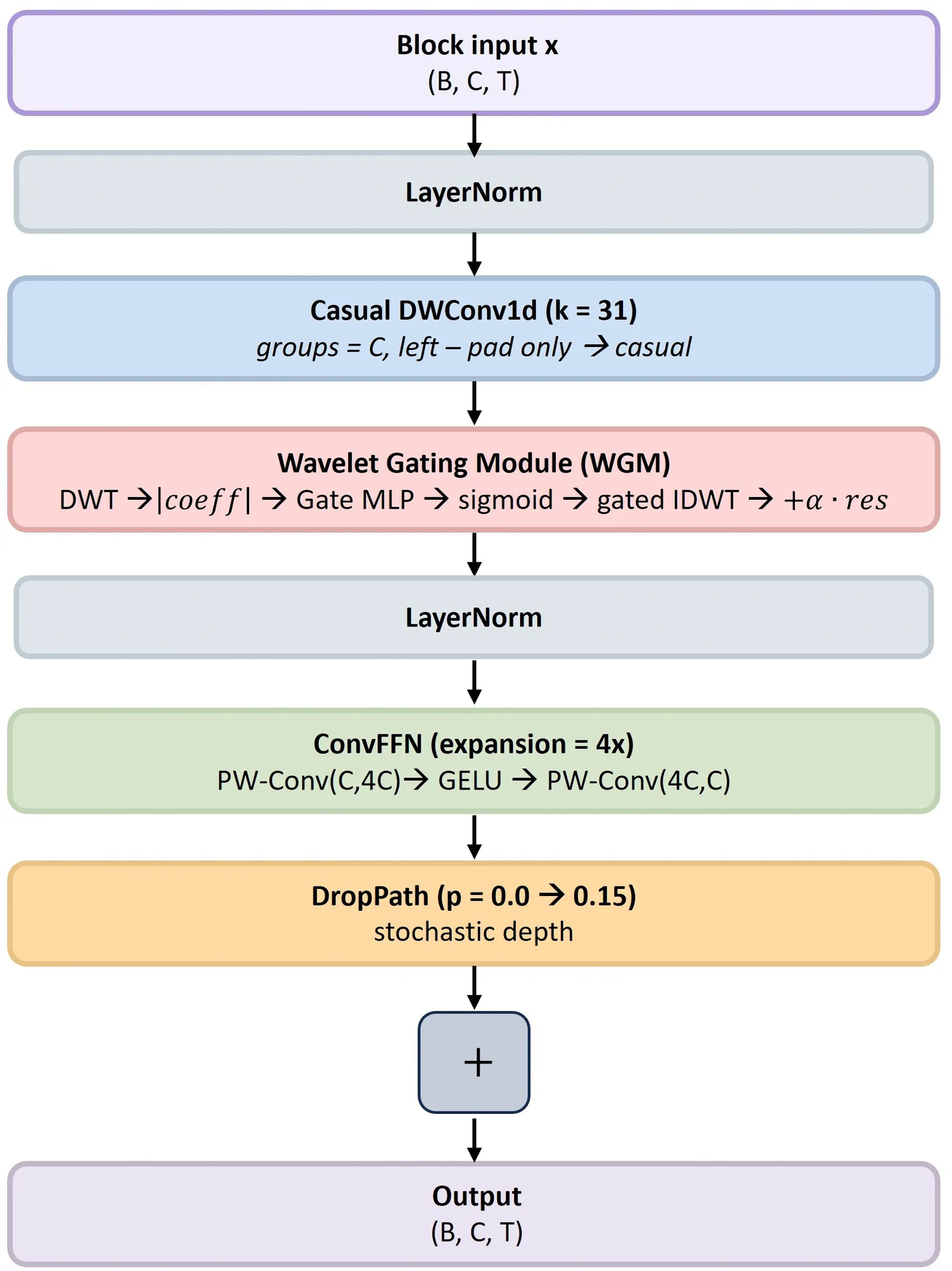
Single ModernTCN block augmented with the proposed WGM.


In the proposed SpectralTCN, we augment this block with the WGM inserted between the depthwise convolution and the ConvFFN, as shown in Fig 3. A single augmented block applies the following transformation:

$$\begin{array}{c} h=x+DropPath\left(ConvFFN\left(LN\left(WGM\left({DWConv}_{k_{s}}\left(CausalPad\left(LN(x)\right)\right)\right)\right)\right)\right) \end{array}$$ (3)

where $x\in R^{B\times d_{s}\times T_{s}}$ is the block input. The four sub-components are described below.

The depthwise convolution processes each channel independently with a large kernel $k_{s}$ = 31:


$$\begin{array}{c} {DWConv}_{k_{s}}(x)=Conv\text{1}d(x;\;groups=d_{s},\;kernel=k_{s}) \end{array}$$ (4)

with causal padding: $k_{s}-\text{1}$ = 30 zeros prepended so that the output at time step t depends only on inputs at times ≤t. The large kernel provides a per-layer receptive field of 31 samples; stacking L blocks yields an effective receptive field of $L(k_{s}-\text{1})+\text{1}$ tokens at the resolution of each stage. Combined with the patch stride of 8 and the three downsampling layers, the receptive field of Stage 3 already spans the entire 1024-sample input window, enabling the network to capture long-range temporal dependencies, such as decaying free-vibration responses, without recurrence or self-attention.

Following the WGM, a two-layer pointwise convolution implements an inverted bottleneck:


$$\begin{array}{c} ConvFFN(x)=W_{\text{2}}\;GELU\left(W_{\text{1}}x+b_{\text{1}}\right)+b_{\text{2}} \end{array}$$ (5)

where $W_{\text{1}}\in R^{rd_{s}\times d_{s}}$ expands the channel dimension by factor r = 4, and $W_{\text{2}}\in R^{d_{s}\times rd_{s}}$ projects it back. Both are implemented as 1×1 convolutions, mixing information across channels without altering the temporal dimension.

To regularise the network, stochastic depth [31] drops each block’s residual branch during training with probability $p_{l}$ that increases linearly from 0 to $p_{\text{max}}$ = 0.2 across all $L=\sum_{s}N_{s}$ blocks:


$$\begin{array}{c} p_{l}=\frac{l}{L-\text{1}}\cdot p_{max},\;l=\text{0},\text{1},\ldots ,L-\text{1} \end{array}$$ (6)

Between consecutive stages, a strided convolution halves the temporal resolution and doubles the channel dimension:


$$\begin{array}{c} {Down}_{s}(x)={Conv\text{1}d}_{k=\text{2},\;s=\text{2}}\left(LN(x)\right)\in R^{B\times d_{s+\text{1}}\times T_{s}/\text{2}} \end{array}$$ (7)

### Wavelet Gating Module (WGM)

The WGM addresses a fundamental limitation of FFT-based spectral processing for SHM: the Fourier transform provides global frequency information with no temporal localisation, yet structural damage often manifests as localised transient events at specific frequency bands and time instants. The DWT provides simultaneous time-frequency resolution and, crucially, each decomposition level corresponds to a specific octave frequency band aligned with structural vibration modes. Because the WGM operates on the patch-embedded feature sequence rather than the raw acceleration signal, its octave bands are defined relative to the token sampling rate. At Stage 1 of the Z24 dataset, the 3-level db2 WGM produces four effective bands: d1 = 3.125–6.25 Hz, d2 = 1.5625–3.125 Hz, d3 = 0.781–1.5625 Hz, and a3 = 0–0.781 Hz.

**Fig 4:**
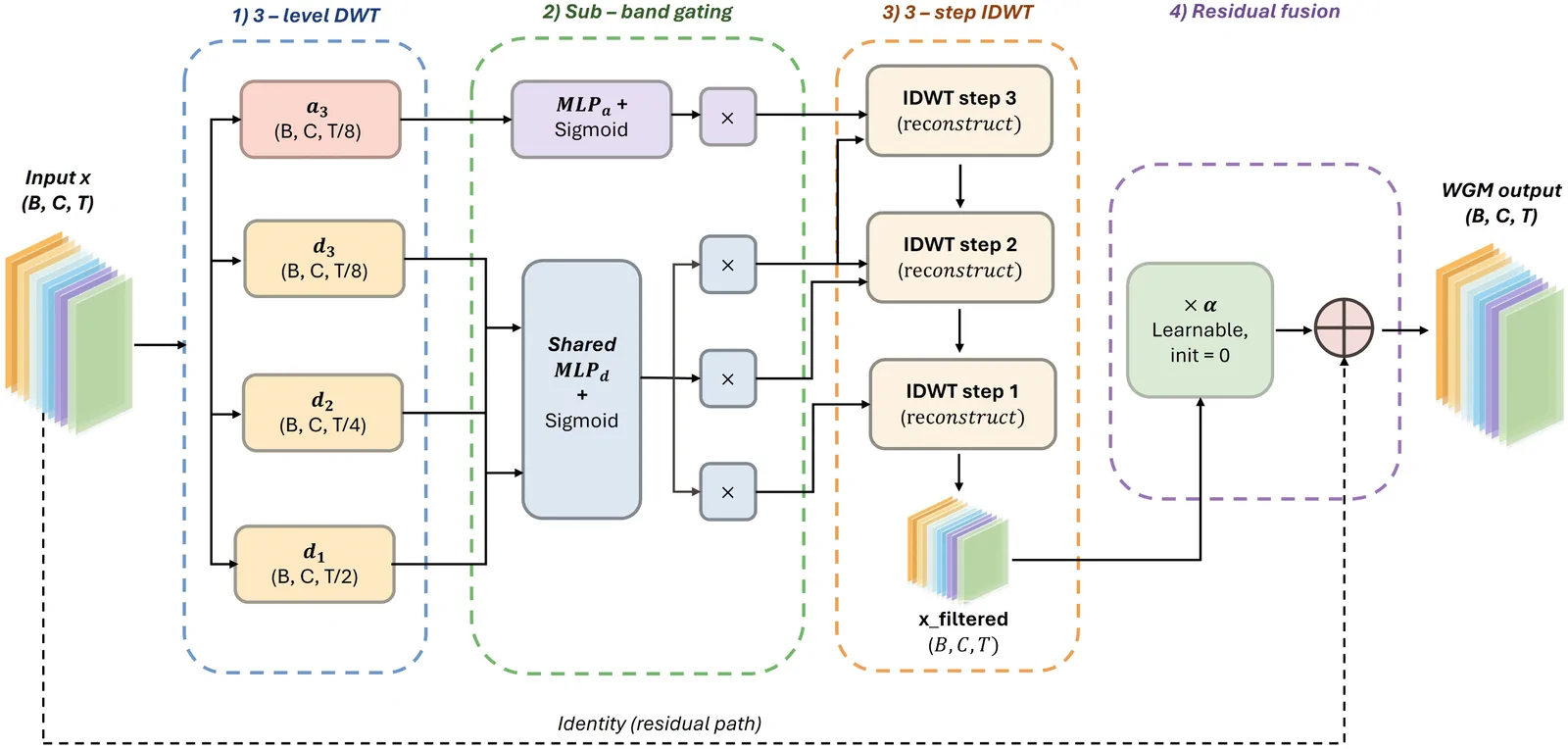
Internal structure of the WGM. Multi-level DWT decomposition produces sub-band coefficients, each modulated by a data-dependent sigmoid gate; the gated coefficients are then reconstructed via the inverse DWT and fused with the input through a learnable residual scalar α initialised to zero.

Given block-internal features $x\in R^{B\times C\times T}$ (output of the causal DWConv), the WGM proceeds in four stages (Fig 4):

Stage 1: Multi-level DWT decomposition. A J-level DWT decomposes x into a set of sub-band coefficient tensors. Each level applies a strided grouped convolution with fixed orthogonal wavelet filters:

$$\begin{array}{c} a_{j+\text{1}}=a_{j}{⊛}_{↓\text{2}}h_{\text{lo}},\;\;d_{j+\text{1}}=a_{j}{⊛}_{↓\text{2}}h_{\text{hi}},\;j=\text{0},\text{1},\ldots ,J-\text{1} \end{array}$$ (8)

where $a_{\text{0}}=x$, ${⊛}_{↓\text{2}}$ denotes strided depthwise convolution with stride 2, and $h_{lo},h_{hi}$ are the low-pass and high-pass decomposition filters of the Daubechies-2 (db2) wavelet. The db2 wavelet is used because it provides compact support and good temporal localisation; its empirical selection is verified in the sensitivity analysis. The filters are fixed (non-trainable) and shared across all channels via grouped convolution. After J = 3 levels, the representation comprises J+1 = 4 sub-band tensors $\left\{a_{J},\;d_{J},\;d_{J-\text{1}},\ldots ,\;d_{\text{1}}\right\}$, each localised to a distinct frequency band.

Stage 2: Data-dependent sub-band gating. A shared two-layer MLP computes a learnable gate for each sub-band independently. For detail level j and for the final approximation $a_{J}$:

$$\begin{array}{c} G_{j}=\sigma {\left(W_{\text{2}}\;GELU\left(W_{\text{1}}\;|d_{j}{|}^{⊤}+b_{\text{1}}\right)+b_{\text{2}}\right)}^{⊤}\in [\text{0},\text{1}{]}^{B\times C\times T/{\text{2}}^{j}} \end{array}$$ (9)

where $W_{\text{1}}\in R^{d_{h}\times C}$, $W_{\text{2}}\in R^{C\times d_{h}}$, $d_{h}=\lfloor \text{0}.\text{5}\;C\rfloor$, and |·| denotes element-wise absolute value. The transpose operation maps $\left(B,C,T_{j})\to (B,T_{j},C\right)$ so that the MLP operates across channels per time step, enabling cross-channel spectral interaction at each resolution. The same MLP weights are shared across all detail levels, so the WGM requires only two gate MLPs – one shared across the J detail sub-bands and one for the approximation sub-band-irrespective of the decomposition depth J. A separate gate MLP is applied to the approximation sub-band $a_{J}$.

Stage 3: Gated IDWT reconstruction. The gated sub-band coefficients are assembled and the signal is reconstructed via the inverse DWT using transposed grouped convolutions:

$$\begin{array}{c} {\tilde{a}}_{j}=\left({\tilde{a}}_{j+\text{1}}⊙G_{a}\right){⊛}^{⊤}h_{lo}+\left(d_{j+\text{1}}⊙G_{j+\text{1}}\right){⊛}^{⊤}h_{hi},\;j=J-\text{1},\ldots ,\text{0} \end{array}$$ (10)

where ${⊛}^{⊤}$ denotes transposed strided convolution (upsampling by factor 2) and ⊙ is element-wise multiplication. For orthogonal wavelets, ConvTranspose1d with the same decomposition filters is the exact adjoint of the analysis step, guaranteeing near-perfect reconstruction when all gates equal unity (empirically verified: maximum reconstruction error $<\text{2}\times {\text{1}\text{0}}^{-\text{7}}$ for db2).

Stage 4: Residual fusion with learnable α**.** The reconstructed signal $x_{filtered}={\tilde{a}}_{\text{0}}$ is fused with the original input via a learnable scalar:

$$\begin{array}{c} WGM(x)=x+\alpha \cdot x_{filtered} \end{array}$$ (11)

where α is initialised to 0. This identity initialisation ensures that at the start of training WGM(x)=x, so the network begins as a vanilla ModernTCN and progressively learns the degree of wavelet filtering required.

The WGM is positioned after the causal DWConv and before the channel-mixing ConvFFN, so that multi-resolution spectral filtering refines per-channel temporal features before they are mixed across channels. Compared with FFT-based gating [11,12], which assigns a single gate per global frequency component, the WGM gate at level j operates on sub-band coefficients of temporal length $T/{\text{2}}^{j}$, preserving both when and at what frequency damage anomalies occur.

### Classification head with Generalized Mean Pooling

The classification head maps the final stage output $x\in R^{B\times d_{S}\times T_{S}}$ to class probabilities. Standard global average pooling treats all temporal positions equally, which may dilute discriminative features that are localised in time (*e.g.*, transient damage signatures). Therefore, GeM pooling [14] is adopted:


$$\begin{array}{c} \text{GeM}(x;\;p)={\left(\frac{\text{1}}{T_{S}}\sum_{t=\text{1}}^{T_{S}}x_{t}^{\;p}\right)}^{\text{1}/p},p\geq \text{1}\;\left(\text{learnable}\right), \end{array}$$ (12)

GeM interpolates smoothly between average pooling (p = 1) and max pooling (p→∞), allowing the network to learn the optimal aggregation strategy from data. The exponent is initialised to $p_{\text{0}}$= 3.0 and clamped to p≥1 during training to ensure numerical stability.

Following pooling, the aggregated feature vector passes through layer normalisation, dropout, and a linear classifier:


$$\begin{array}{c} \hat{y}=W_{cls}\;Dropout\left(LN(v)\right)+b_{cls}\;\in \;R^{B\times K}, \end{array}$$ (13)

where $W_{\text{cls}}\in R^{K\times d_{S}}$, K is the number of structural states, and the dropout rate is 0.1. During inference, a softmax is applied to obtain class probabilities.

## Experiments

### Dataset

#### Z24 bridge benchmark.

**Fig 5:**
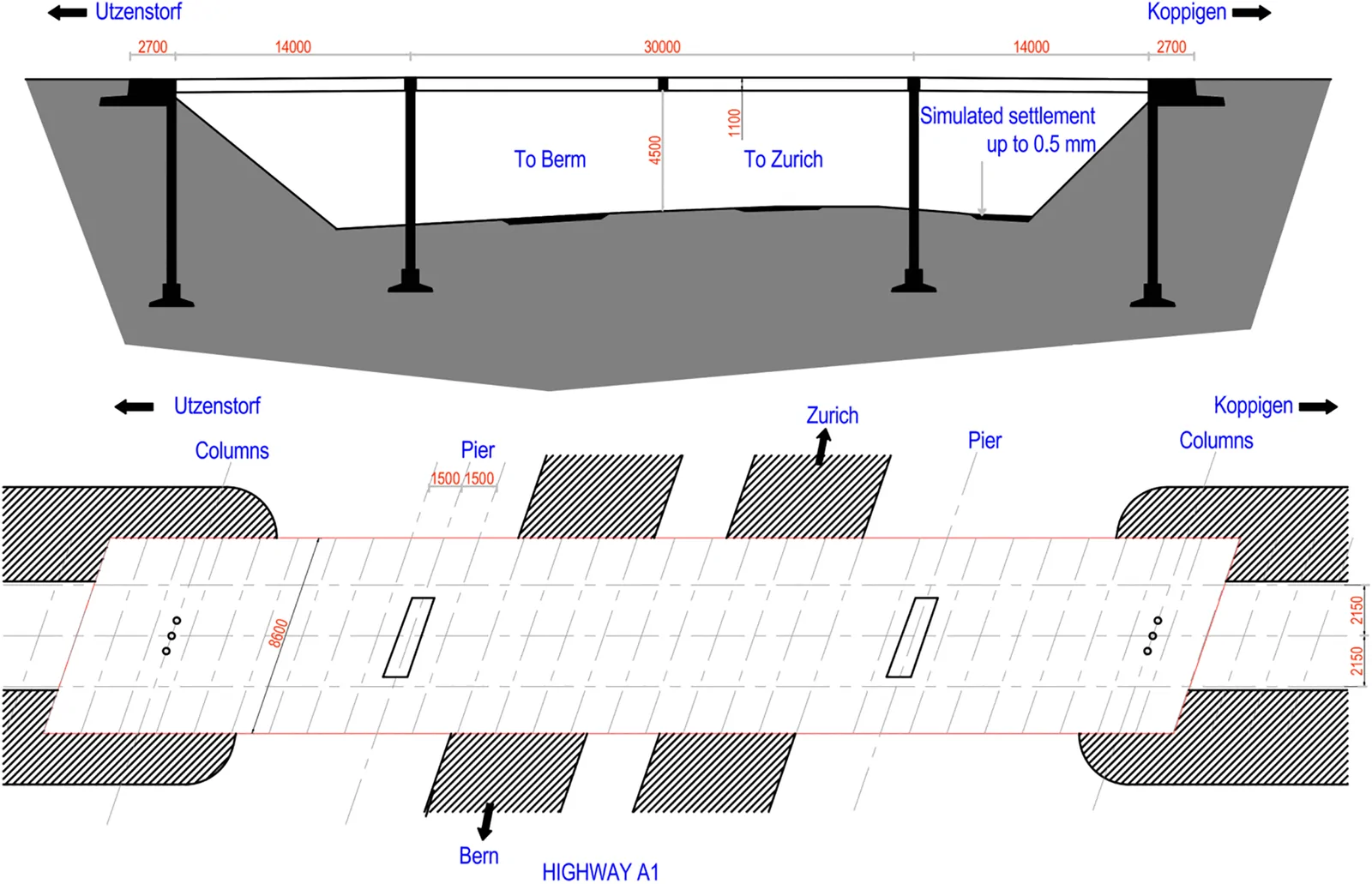
General view of the Z24 Bridge, Switzerland [15].

The Z24 Bridge (Fig 5) is a post-tensioned concrete highway bridge in Switzerland that was monitored continuously for 10 months (November 1997–September 1998) before controlled demolition [15]. The monitoring system comprised 27 accelerometers recording at 100 Hz. The dataset includes 17 structural states: two reference conditions (Classes 0 and 7) and 15 progressively applied damage scenarios covering pier settlement and lifting, foundation tilt, abutment landslide, concrete spalling, concrete-hinge failure, anchor-head failure and tendon rupture, as summarised in Table 1**.** This diversity of damage types at varying severity levels makes it one of the most widely used benchmarks for multi-class vibration-based SHM classification.

**Table 1:** Damage scenarios on the Z24 Bridge [15].

Class	Date (1998)	Description	Class	Date (1998)	Description
0	4 Aug.	Undamaged condition	9	26 Aug.	Spalling of concrete at soffit, 24 m^2^
1	9 Aug.	Installation of pier settlement system	10	27 Aug.	Landslide of 1 m at abutment
2	10 Aug.	Lowering of pier, 20 mm	11	31 Aug.	Failure of concrete hinge
3	12 Aug.	Lowering of pier, 40 mm	12	2 Sep.	Failure of 2 anchor heads
4	17 Aug.	Lowering of pier, 80 mm	13	3 Sep.	Failure of 4 anchor heads
5	18 Aug.	Lowering of pier, 95 mm	14	7 Sep.	Rupture of 2 out of 16 tendons
6	19 Aug.	Lifting of pier, tilt of foundation	15	8 Sep.	Rupture of 4 out of 16 tendons
7	20 Aug.	New reference condition	16	9 Sep.	Rupture of 6 out of 16 tendons
8	25 Aug.	Spalling of concrete at soffit, 12 m^2^			

**Fig 6:**
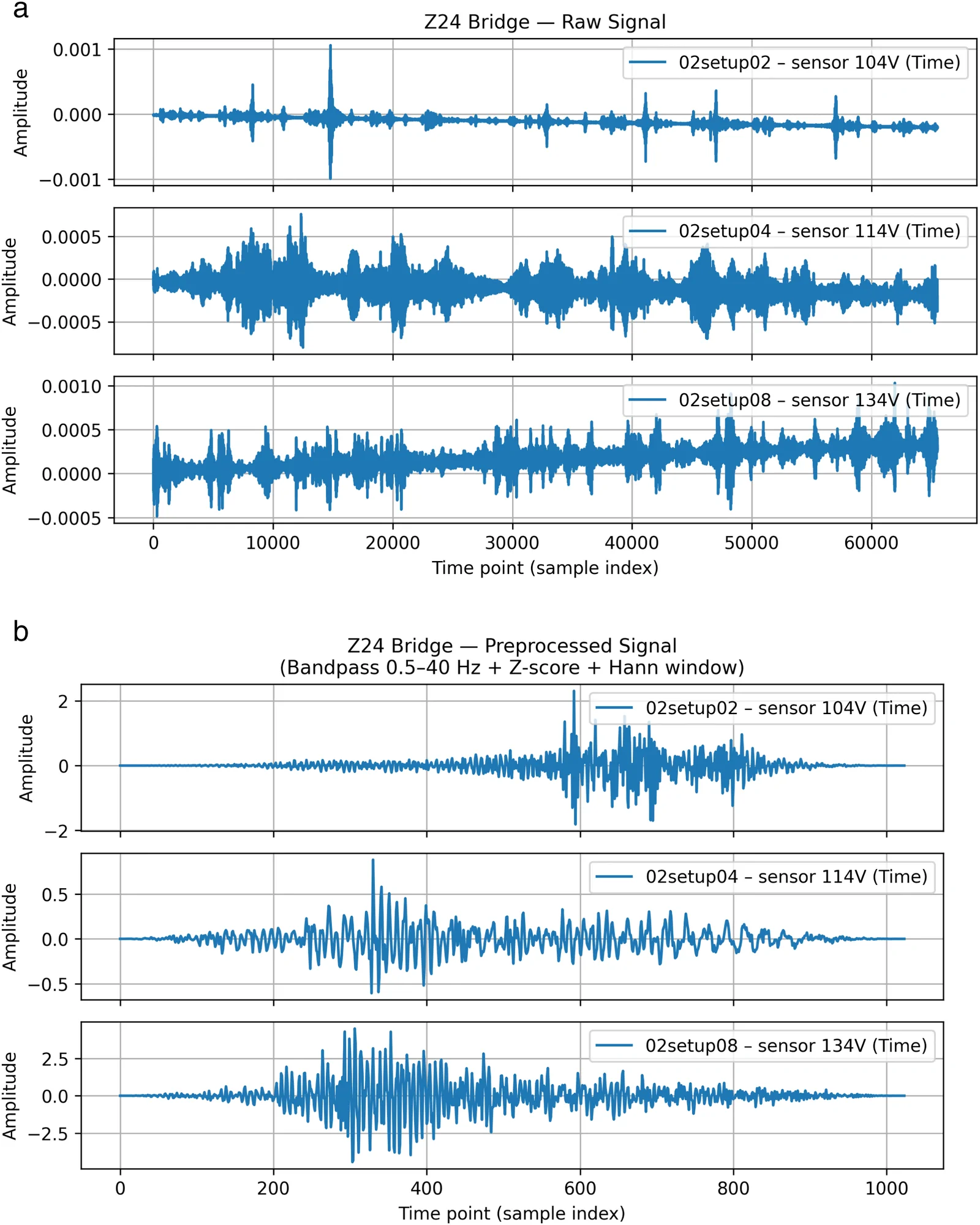
Representative acceleration signals from the Z24 Bridge dataset. (a) Raw recordings; (b) after bandpass filtering (0.5–40 Hz), z-score normalisation, and Hann windowing.

Raw signals were preprocessed with a 4^th^-order zero-phase Butterworth bandpass filter (0.5–40 Hz), which effectively detrends the signals by eliminating DC offsets and low-frequency drifts, followed by per-channel z-score normalisation, and sliding-window segmentation using a Hann window of 1024 samples (≈10.24 s), as illustrated in Fig 6.

The resulting dataset comprises 15,300 windows of shape (27, 1024), partitioned by stratified 5-fold cross-validation into approximately 9,940 training, 2,300 validation, and 3,060 test samples per fold. As a real-world monitoring dataset, the Z24 acceleration records also retain environmental and operational variability, including ambient excitation, traffic-induced vibration, temperature effects, loading variation, and sensor noise. Therefore, the Z24 benchmark provides a naturally noisy field-measurement setting rather than an idealised clean-signal dataset.

#### My Thuan cable-stayed bridge (FEM dataset).

**Fig 7:**
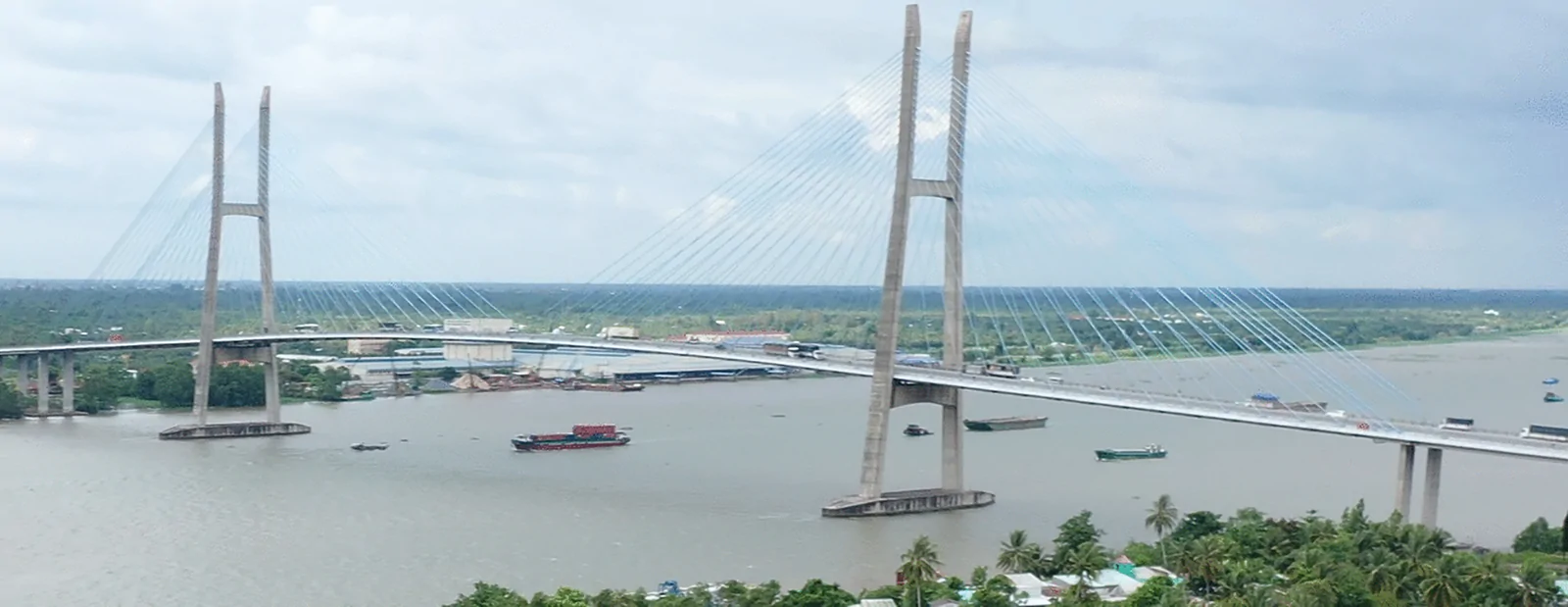
My Thuan cable-stayed bridge, Vinh Long Province, Vietnam.

**Fig 8:**
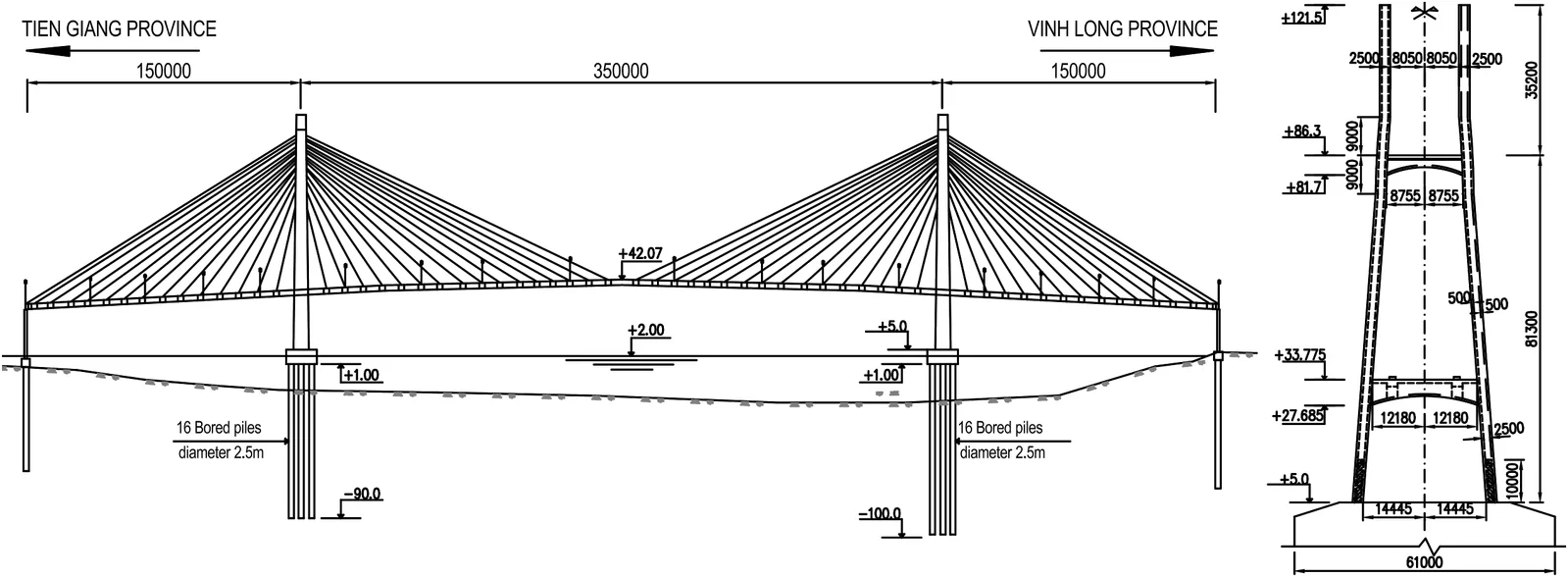
General view and tower section of the My Thuan cable-stayed bridge.

The My Thuan Bridge spans the Tien River in Vinh Long Province, Vietnam, with a total length of 1,535 m (Fig 7). The main bridge is a cable-stayed structure comprising three spans of 150–350–150 m. The two towers are modified H-frame pylons made of grade 50 reinforced concrete, rising 123.5 m above the pile cap and 84.43 m above the deck level; each tower is supported by a foundation of 16 bored piles with a diameter of 2.5 m (Fig 8). The stay-cable system consists of two vertical cable planes arranged along both sides of the main girder, symmetric about the bridge centreline and separated by 18.6 m, with a fan-shaped layout comprising 4 × 32 = 128 cables in total, arranged as four fans of 32 cables, two at each tower. The deck is 23.6 m wide and 0.25 m thick, accommodating four vehicle lanes and two pedestrian walkways. It is supported by two longitudinal grade 50 reinforced-concrete girders of 1.76 m height, together with transverse beams spaced at 5.2 m intervals.

**Fig 9:**
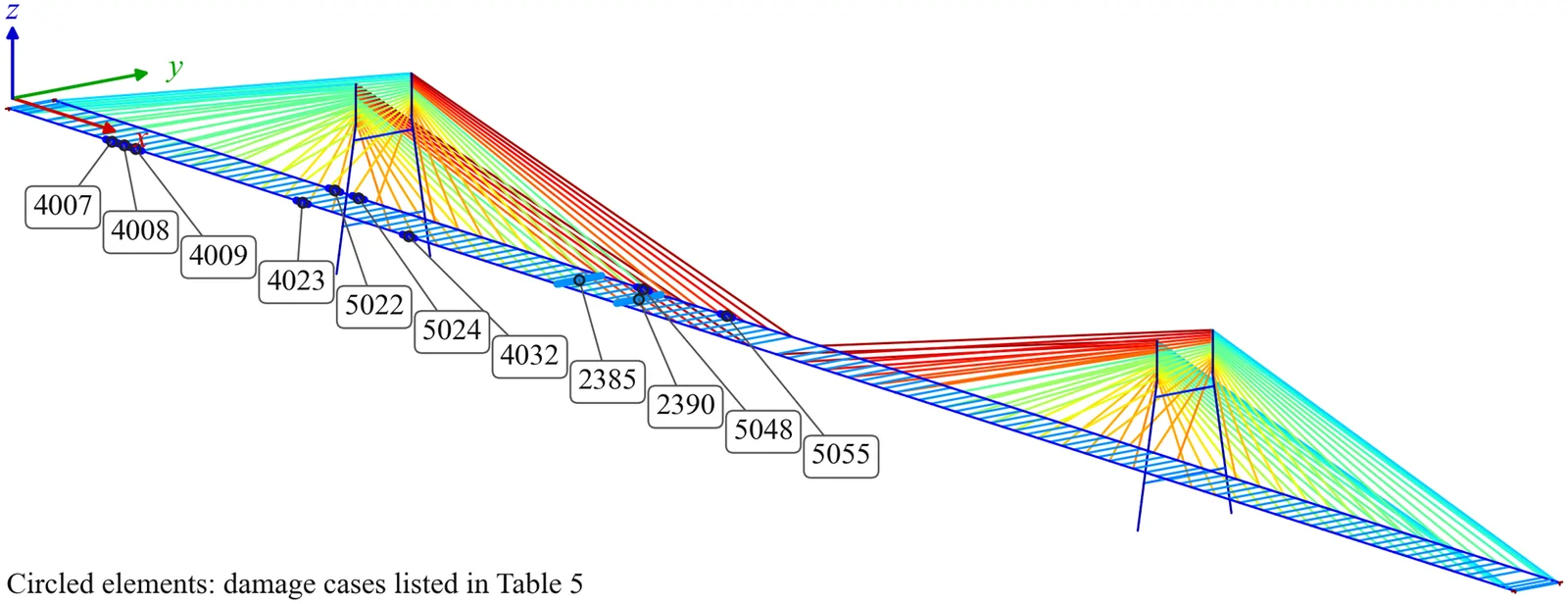
The FEM of the My Thuan cable-stayed bridge used to generate the simulation dataset.

While the Z24 dataset provides critical validation for real-world applicability, executing severe or progressive structural damage scenarios on in-service infrastructure is prohibited due to safety constraints. To overcome this limitation and evaluate the model’s capability to detect granular levels of structural deterioration, a FEM of the My Thuan bridge (Fig 9) was developed. This controlled simulation setting serves as a complementary benchmark.

The FEM of the My Thuan cable-stayed bridge is built in MATLAB using the Stabil Toolbox. The X-axis denotes the longitudinal direction, Y the transverse direction, and Z the vertical direction. The model is discretised into a three-dimensional truss-beam system comprising the deck, transverse beams, towers, stay cables and so on. Stay cables are modelled as truss elements whose prestress is represented through equivalent material constants. The main load-carrying members and stay cables are modelled as steel ($E=\text{2}.\text{1}\text{4}\text{0}\text{1}\times {\text{1}\text{0}}^{\text{1}\text{1}}$ Pa, ν = 0.3, ρ = 7810 kg/m3), while concrete components (main girder, deck slab, cross beams, towers) use lower stiffness values reflecting their respective grades. The cross-sectional properties of all structural elements are listed in Table 2, and the measured tension forces and lengths of the 32 cables of one representative fan are given in Table 3. The material properties were calibrated through model updating using ambient vibration measurements [32]; the corresponding reduction in modal frequency prediction error is reported in Table 4.

**Table 2:** Cross-sectional characteristics of the My Thuan bridge.

No.	Element		Cross-sectional Area (mm2)	Moment of Inertia $I_{z}$ (mm4)	Moment of Inertia $I_{y}$ (mm4)
1	Main girder		3.53×10^6^	1.49 × 10^12^	3.29 × 10^12^
2	Cross beam		9.38 ×10^6^	3.70 × 10^12^	5.20 × 10^10^
3	Bridge deck		3.70 ×10^6^	1.93 × 10^12^	6.54 × 10^13^
4	Tower	Section 1	7.05×10^6^	4.51 × 10^12^	1.20 × 10^13^
		Section 2	7.04 ×10^6^	6.24 × 10^12^	2.85 × 10^13^
		Section 3	9.57×10^6^	1.91 × 10^12^	2.11 × 10^13^
		Section 4	3.30×10^6^	2.27 × 10^12^	2.51 × 10^13^
		Section 5	7.57×10^6^	1.91 × 10^12^	2.11 × 10^13^

**Table 3:** Measured tension forces (F, kN) and lengths (L, m) of the 32 stay cables in one representative fan of the My Thuan bridge.

No.	F	L	No.	F	L	No.	F	L	No.	F	L
1	6395	177.5	9	3266	114.3	17	2512	57.4	25	2947	118.3
2	5264	173.8	10	3128	105.2	18	2376	63.4	26	3145	127.6
3	4554	170.4	11	2935	96.4	19	1938	69.6	27	3417	137.1
4	3668	162.4	12	2543	88.0	20	2135	76.4	28	3688	146.8
5	3459	152.5	13	2267	80.0	21	2508	83.9	29	4077	156.5
6	3459	142.8	14	2497	72.6	22	2743	91.9	30	4006	165.4
7	3481	133.1	15	2042	65.5	23	2403	100.4	31	4489	176.3
8	3478	123.6	16	2171	58.5	24	3033	109.2	32	5967	186.4

**Table 4:** Modal frequencies of the My Thuan bridge before and after model updating [32].

Mode	Before (Hz)	After (Hz)	Experimental (Hz)	Error before (%)	Error after (%)
Mode 1	0.36	0.38	0.39	7.7	2.6
Mode 2	0.56	0.59	0.59	5.1	0.0
Mode 3	0.59	0.63	0.65	9.2	3.2
Mode 4	0.67	0.70	0.71	5.6	1.4

Before generating the training dataset, the FE model is calibrated against the in-situ behaviour of the bridge. An ambient vibration measurement campaign was conducted to extract the operational modal parameters (natural frequencies and mode shapes) using the Stochastic Subspace Identification (SSI) method. Comparison between the initial FE model and the experimental measurements revealed notable discrepancies, primarily attributable to uncertainties in the elastic moduli of the concrete elements, the cable prestress force, and the boundary conditions at the supports. These uncertain parameters are updated through a hybrid algorithm combining a Genetic Algorithm (GA) with an Improved Particle Swarm Optimisation (HGAIPSO) [32], yielding the calibrated model that serves as the reliable baseline for generating the synthetic damage scenarios. Table 4 reports the first four modal frequencies together with their errors relative to experiment.

The synthetic dataset covers 11 distinct structural states, comprising one healthy reference (Case 0) and ten damage scenarios (Cases 1–10). Damage is simulated by reducing the stiffness of specific beam members, achieved by lowering the Young’s modulus E in the finite-element formulation. The damaged stiffness is computed as


$$\begin{array}{c} E_{damaged}=E_{initial}\times (\text{1}-\alpha ) \end{array}$$ (14)

where α is the damage severity. The damage scenarios involve stiffness reductions ranging from 10% to 40% applied to considered members. For every damaged member, a new material row carrying the reduced E is appended to the material matrix and reassigned to the target element. The complete list of the 11 cases (one undamaged reference and ten damage scenarios) is summarised in Table 5.

**Table 5:** Damage cases applied to the My Thuan bridge FEM.

Label	Element	Damage (%)	State
0	4007	0	Undamaged
1	4008	10	Damage
2	4009	20	
3	4023	30	
4	5022	40	
5	5024	30	
6	4032	10	
7	2385	20	
8	2390	10	
9	5048	30	
10	5055	10	

To reproduce realistic operational conditions, the bridge is subjected to a moving load representing a two-axle truck consistent with AASHTO standards: the front axle carries 35 kN and the rear axle 145 kN, with the axles spaced 4.3 m apart. The truck travels at a constant speed of 60 km/h (≈16.67 m/s) and crosses the bridge 8 consecutive times with a 3 s gap between successive passes. To account for ambient excitation between vehicle passes, an impulsive force (−5000 N), wind effects, local-traffic noise, and a periodic component driven by the structural natural frequencies are superposed. The dynamic response is solved by modal superposition in the frequency domain with 20 modes retained and a modal damping ratio of ξ= 1.5%, then transformed back to the time domain through the inverse FFT. These periodic and Gaussian noise components were added as low-amplitude operational perturbations to make the FEM responses closer to realistic measurements, rather than as class-specific cues. The damage labels are still defined by stiffness reductions in selected members, with class differences mainly reflected in the modal-frequency content of the acceleration signals.

The structural response is recorded by extracting the vertical acceleration of the lower-beam nodes belonging to the two side lanes (node IDs 405–592 for the left lane and 605–792 for the right lane), sampled at uniform spacing along each lane, yielding 36 virtual sensors in total. Acceleration time histories are obtained by twice differentiating the nodal displacement using a central-difference scheme, with a sampling interval dt= 0.002 s (500 Hz) and a simulation window of T≈109.6 s, producing approximately N≈ 54,780 time points per signal. The complete raw dataset is therefore organised as an array of size (11, 36, ≈ 54,780).

**Fig 10:**
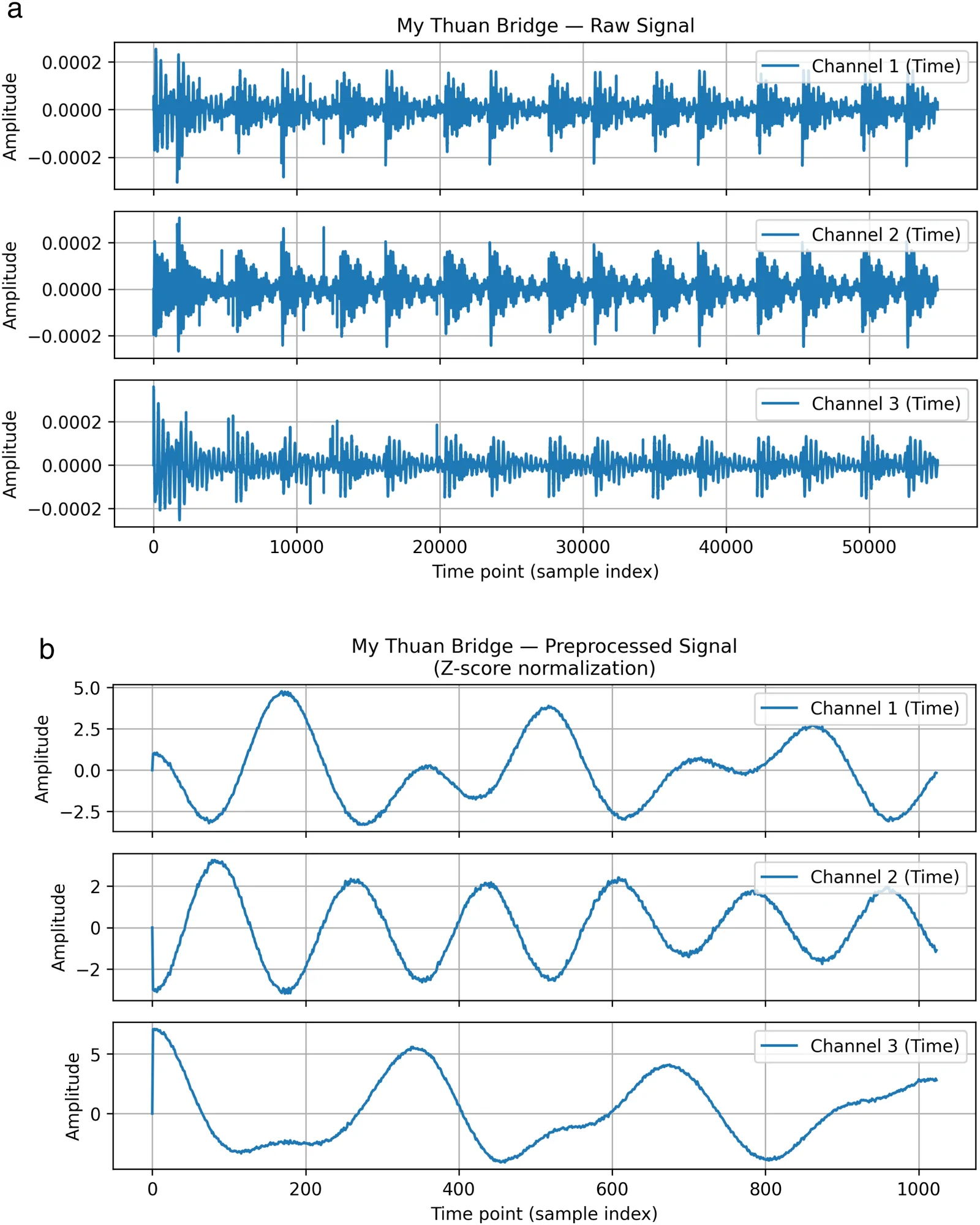
Representative acceleration signals from the My Thuan Bridge FEM dataset (healthy state). (a) Raw simulated signals; (b) after z-score normalisation.

For the deep learning pipeline, the raw signals are then preprocessed with per-channel z-score normalisation and rectangular sliding-window segmentation of 1024 samples, as illustrated in Fig 10.

The resulting dataset contains 4,532 windows of shape (36, 1024), partitioned by stratified 5-fold cross-validation into approximately 2,937 training, 671 validation, and 924 test samples per fold.

#### Class balance and data splitting.

Class balance and leakage prevention were explicitly considered for both datasets. Although the original Z24 recordings have different durations across structural states, class balance was controlled during preprocessing by retaining an equal-length segment of 60,000 time points per channel for each state before sliding-window segmentation. For the My Thuan FEM dataset, acceleration responses were generated with the same signal duration for all simulated structural states; therefore, the resulting windows were naturally balanced across classes. Consequently, no class weighting, oversampling, or undersampling was applied during training.

For the Z24 dataset, stratified 5-fold cross-validation was performed at the level of complete measurement records rather than at the window level. Each record was assigned entirely to one fold, and sliding-window segmentation was applied only after the split within the training, validation, and test partitions. Therefore, no window extracted from the same measurement record could appear in both the training and test sets.

For the My Thuan FEM dataset, because each structural state contains one long simulated recording, a record-level split was not applicable. Instead, stratified 5-fold cross-validation was performed using temporal blocking within each recording. In each fold, a distinct contiguous segment from every state was held out for testing, and a purge gap equal to the window-overlap span was removed at each test boundary to ensure that no training window shared raw samples with the test windows. Normalisation statistics were fitted using only the training windows in all cases.

### Implementation details and evaluation metrics

#### Implementation environment and architecture configuration.

All experiments were conducted on a Windows 11 (64-bit) workstation equipped with an Intel Core i9-13900HX processor (24 cores/ 32 threads, up to 5.4 GHz on performance cores, 36 MB cache) and an NVIDIA GeForce RTX 4060 GPU with 8 GB VRAM. All models were implemented in Python 3.11 using PyTorch 2.7 with CUDA 11.8 for GPU-accelerated training.

To ensure reproducibility and a fair comparison, the identical SpectralTCN configuration was applied to both datasets. The complete set of architectural, optimisation, and regularisation hyperparameters is detailed in Table 6.

**Table 6:** Hyperparameter configurations of the proposed SpectralTCN framework.

Category	Hyperparameter	Selected value	Description
SpectralTCN Backbone	Hierarchy stages	4	Hierarchical temporal feature extraction
	Block depths	[2, 2, 8, 2]	Number of blocks in each stage
	Channel dimensions	[48, 96, 192, 384]	Feature dimension of each stage
Temporal convolutional	Depthwise convolutional kernel size	31	Captures temporal dependencies
Patch embedding	Patch size	8	Converts the input signal into temporal patches
	Stem kernel	15	Initial local temporal feature extraction
ConvFFN	Expansion Ratio(r)	4	Hidden expansion ratio in the feed-forward module
Wavelet-Gated Module	Mother wavelet	Daubechies – 2, db2	Mother wavelet used for DWT decomposition
	Decomposition level	3	Multi-resolution wavelet decomposition level
	Gate MLP hidden ratio	0.5	Hidden ratio of the gating MLP
	Residual scalar α	0, learnable	Controls the residual contribution of the wavelet branch
Pooling	GeM exponent p	3.0, learnable	Initial exponent of generalized mean pooling
Regularisation	Maximum DropPath rate	0.2	Stochastic depth regularisation
	Head dropout	0.1	Dropout before the classification head
Optimisation	Optimiser	AdamW	Weight-decoupled adaptive optimiser
	Learning rate	1 × 10^−3^	Initial learning rate
	Weight decay	0.05	Regularisation term in AdamW
Training	Batch size	128	Mini-batch size
	Maximum epochs	100	Maximum number of training epochs
	Early stopping patience	15 epochs	Early stopping based on validation performance

Regarding the WGM, the hyperparameters were selected based on prior vibration signal analysis studies and preliminary validation. The Daubechies-2 (db2) mother wavelet was adopted for its compact support and suitability for representing localised signal variations, which was further confirmed via sensitivity analysis. A 3-level decomposition was chosen to provide a balanced multi-resolution representation without excessively increasing computational complexity or over-smoothing the temporal features. Finally, all models were optimised using AdamW and trained for up to 100 epochs with early stopping.

#### Evaluation metrics.

**Fig 11:**
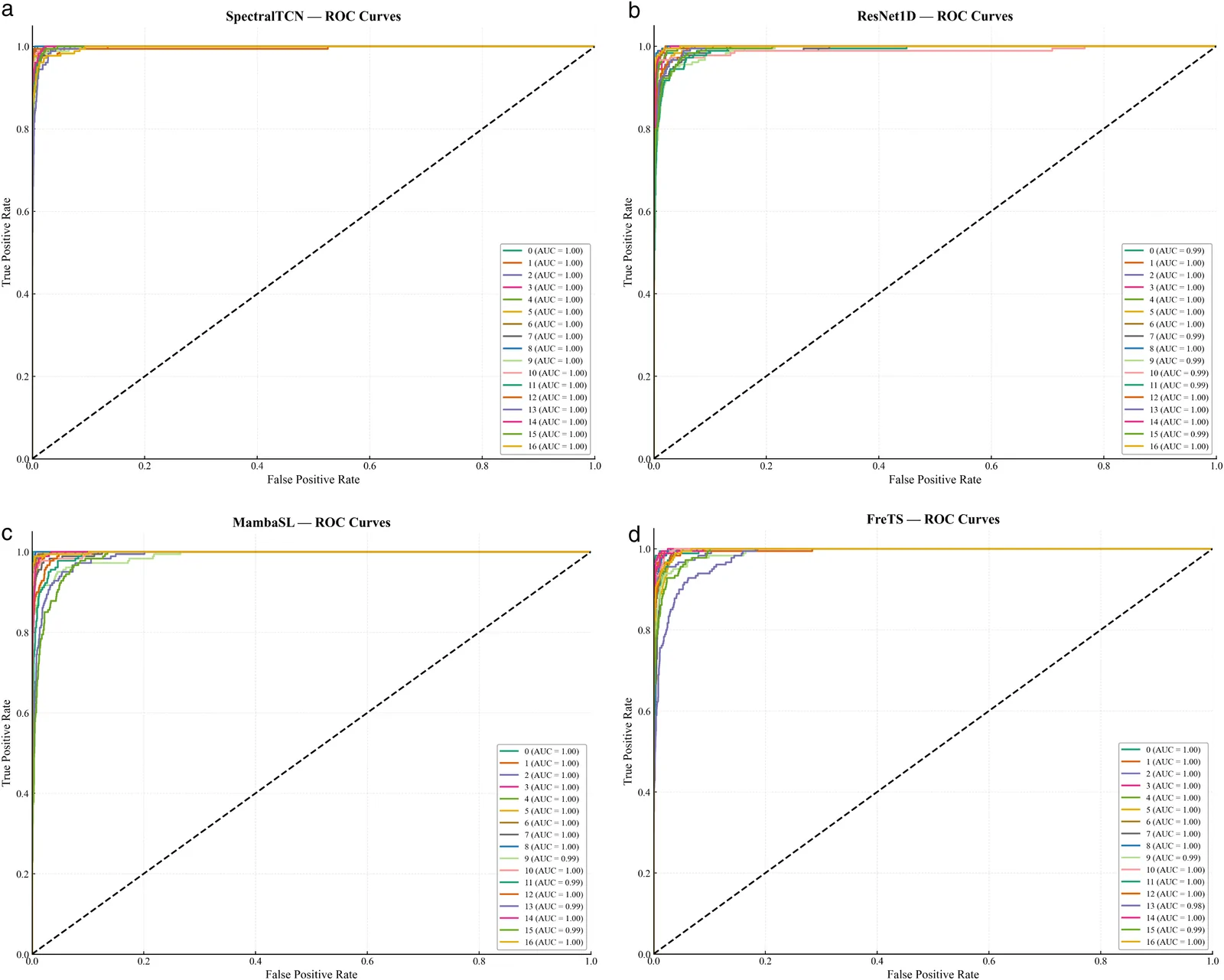
Macro-averaged ROC-AUC curves (one-versus-rest) on the Z24 Bridge for the four best-performing models in fold 4. Each curve corresponds to one of the 17 structural states. (a) SpectralTCN; (b) ResNet1D; (c) MambaSL; (d) FreTS.

Six metrics are used to assess multi-class classification performance. Accuracy, F1-macro and MCC are reported in Tables 7 and 16, per-class Precision and Recall in Tables 15 and 19, and macro ROC-AUC in Fig 11, Fig 19 and Table 8. Let TP, TN, FP, and FN denote the numbers of true positives, true negatives, false positives, and false negatives, respectively. For multi-class problems, each metric is computed per class in a one-versus-rest manner and then averaged.

**Precision** measures the fraction of positive predictions that are correct:


$$\begin{array}{c} Precision=\frac{TP}{TP+FP} \end{array}$$ (15)

**Recall** (sensitivity) measures the fraction of actual positives that are correctly identified:


$$\begin{array}{c} Recall=\frac{TP}{TP+FN} \end{array}$$ (16)

**F1-score** is the harmonic mean of Precision and Recall, balancing both aspects:


$$\begin{array}{c} F\text{1}=\frac{\text{2}\times Recall\times Precision}{Recall+Precision} \end{array}$$ (17)

**Accuracy** is the overall fraction of correctly classified samples:


$$\begin{array}{c} Accuracy=\frac{TP+TN}{TP+TN+FP+FN} \end{array}$$ (18)

**Matthews Correlation Coefficient** (MCC) provides a balanced measure robust to class imbalance:


$$\begin{array}{c} MCC=\frac{TP\times TN-FP\times FN}{\sqrt{\left(TP+FP)(TP+FN)(TN+FP)(TN+FN\right)}} \end{array}$$ (19)

**ROC-AUC** (macro-averaged, one-versus-rest) summarises classifier discrimination across all damage classes. All metrics are computed on the held-out test partition of each of the 5 folds of the stratified cross-validation and reported as the mean ± standard deviation.

To rigorously assess the trade-off between classification performance and computational efficiency for near-real-time SHM deployment, we evaluate all models not only on accuracy metrics but also on their computational cost, specifically the number of trainable parameters (Params) and Multiply-Accumulate operations per window (MACs/window).

### Compared methods

We compare SpectralTCN against 10 baseline models grouped into four representative families:

- **Convolution-based models:** 1DCNN [5], ResNet1D [33], TCN [8]
- **Recurrent-based models:** GRU [34], 1DCNN-LSTM [35]
- **Transformer and MLP-based models:** Transformer [7], PatchTST [36], TSMixer [37]
- **Frequency and state-space models**: FreTS [12], MambaSL [28]

All baselines are trained with the same preprocessing pipeline, data splits, training budget, optimisation strategy, and evaluation protocol to ensure a fair comparison.

### Results on Z24 Bridge

#### Comparison with baseline models.

Table 7 summarises the classification performance of all models on the Z24 Bridge. SpectralTCN achieves the highest accuracy of 0.922 ± 0.015, outperforming all baselines on all three metrics. Among the baselines, ResNet1D ranks second at 0.904 ±0.007, followed closely by MambaSL (0.897 ± 0.007) and FreTS (0.894 ± 0.005). These four models form a top cluster (accuracy ≥ 0.89), all of which either leverage multi-scale temporal features (ResNet1D), state-space sequence modelling (MambaSL), or global spectral processing (FreTS), suggesting that either multi-scale temporal modelling or explicit spectral processing is helpful for the 17-class Z24 problem. SpectralTCN improves over ResNet1D by 1.8 percentage points in accuracy and 1.9 in MCC, a consistent margin that persists across all five folds (standard deviation of ± 0.015), suggesting that wavelet-domain multi-resolution filtering provides a complementary inductive bias beyond purely residual temporal feature extraction. SpectralTCN therefore attains the best classification performance at a moderate inference cost of 152.62 M MACs per window, below ResNet1D (3460.11 M) and PatchTST (1355.36 M), although it also has the largest parameter count (6.47 M) among the compared models.

**Table 7:** Classification performance on the Z24 Bridge (5-fold CV, mean ± std, 17 classes). Best in bold, second best underlined.

Model	Param (M)	Accuracy	F1-macro	MCC	MACs/window (M)
1DCNN	0.16	0.433± 0.063	0.423 ± 0.075	0.410 ± 0.053	29.17
ResNet1D	3.39	0.904 ±0.007	0.904 ± 0.008	0.898 ± 0.008	**3460.11**
TCN	0.19	0.843 ± 0.029	0.842 ± 0.031	0.833 ± 0.033	208.34
1DCNN-LSTM	0.72	0.854 ± 0.008	0.854 ± 0.007	0.845 ± 0.009	201.13
Transformer	0.85	0.868 ± 0.005	0.868 ± 0.005	0.860 ± 0.006	37.10
PatchTST	0.62	0.607± 0.023	0.605 ± 0.023	0.583 ± 0.022	1355.36
TSMixer	4.26	0.865 ± 0.014	0.864 ± 0.015	0.857 ± 0.015	169.87
MambaSL	0.17	0.897 ± 0.007	0.898 ± 0.007	0.891 ± 0.008	20.93
FreTS	0.28	0.894 ± 0.005	0.894 ± 0.006	0.888 ± 0.005	33.69
GRU	0.42	0.812 ± 0.007	0.813 ± 0.007	0.801 ± 0.007	105.98
**SpectralTCN**	**6.47**	**0.922** ± **0.015**	**0.922** ± **0.017**	**0.917** ± **0.017**	152.62

Notably, FreTS, which uses FFT-based global spectral gating, achieves 0.894 ±0.005, whereas SpectralTCN, which applies DWT-based time-localised gating inside a ModernTCN backbone, reaches 0.922 ± 0.015, a gap of 2.8 percentage points. Because FreTS and SpectralTCN differ in more than the choice of transform, this gap should be read as an architecture-level comparison rather than a controlled DWT-versus-FFT test. The controlled comparison is the A2-versus-A3 ablation in Table 10, where replacing the FFT gate with the WGM inside an identical backbone yields a smaller gain of 0.3 percentage points. Both observations are consistent with, but do not by themselves establish, the hypothesis that the temporal localisation preserved by the DWT is helpful for detecting transient, frequency-localised damage signatures. Models designed primarily for time-series forecasting (PatchTST: 0.607 ± 0.023) or shallow temporal convolution (1DCNN: 0.433 ± 0.063) perform substantially below the leading group, consistent with the expectation that deeper, multi-scale architectures are better suited to the fine-grained 17-class discrimination required by the Z24 benchmark. The ROC-AUC curves in Fig 11 further illustrate the separation quality: SpectralTCN achieves consistently high one-versus-rest discrimination across nearly all 17 structural states, whereas ResNet1D and MambaSL exhibit slightly wider variance at the lower-severity damage states.

Following the performance comparison and ROC-AUC analysis, the statistical reliability of the observed improvements was assessed using fold-wise cross-validation results. Tables 8 and 9 report pairwise tests between SpectralTCN and the strongest baseline, ResNet1D, together with Friedman tests across all compared models.

**Table 8:** Pairwise comparison between SpectralTCN and the strongest competing baseline, ResNet1D.

Metric	SpectralTCN	ResNet1D	Mean difference	Paired t-test p-value	Wilcoxon p-value
Accuracy	92.22 ± 1.53 [90.32, 94.12]	90.35 ± 0.73 [89.45, 91.25]	+ 1.87	0.0715	0.0312
F1-macro	92.20 ± 1.58 [90.24, 94.16]	90.38 ± 0.71 [89.51, 91.26]	+ 1.82	0.0796	0.0312
MCC	91.75 ± 1.62 [89.73, 93.76]	89.77 ± 0.77 [88.82, 90.72]	+ 1.98	0.0726	0.0312
ROC-AUC macro	99.76 ± 0.12 [99.62, 99.90]	99.66 ± 0.10 [99.54, 99.78]	+ 0.10	0.0138	0.0312

**Table 9:** Friedman test across all 11 compared models. Values are reported as mean ± standard deviation with 95% confidence intervals. Mean differences are reported in percentage points. Wilcoxon p-values are one-sided. Lower Friedman mean rank indicates better average performance across folds.

Metric	Friedman	p-value	SpectralTCN mean rank	ResNet1D mean rank
Accuracy	43.376	< 0.001	1.200	2.800
F1-macro	43.236	< 0.001	1.200	2.800
MCC	43.273	< 0.001	1.200	2.800
ROC-AUC macro	42.182	< 0.001	1.400	3.600

As shown in Table 9, the Friedman tests indicate statistically significant differences among the compared models for all metrics, while the Wilcoxon tests show consistent fold-wise improvements of SpectralTCN over ResNet1D.

#### Ablation study.

Table 10 reports the Z24 ablation results. The ModernTCN backbone alone (A1) achieves 0.876 ± 0.052 test accuracy, with the large standard deviation (± 0.052) reflecting sensitivity to fold composition in the absence of any spectral regularisation. Adding FFT-based global spectral gating (A2) raises accuracy to 0.914 ± 0.018 and markedly reduces the validation loss from 0.389 to 0.247, indicating that explicit frequency-domain processing benefits the backbone.

**Table 10:** Ablation study on the Z24 Bridge (5-fold CV, mean ± std)*.* Best result in bold.

Variant	Param(M)	Val Loss	Val Acc	Test Acc	MCC
A1: ModernTCN backbone	4.63	0.389 ± 0.203	0.881 ± 0.073	0.876 ± 0.052	0.868 ± 0.055
A2: A1 + FFT	5.17	0.247 ± 0.071	0.924 ± 0.025	0.914 ± 0.018	0.909± 0.019
A3: A1 + WGM	5.71	0.227 ± 0.051	0.927 ± 0.015	0.917 ± 0.017	0.912 ± 0.018
A4: A1 + GeM	4.63	0.282 ± 0.075	0.919 ± 0.012	0.913 ± 0.012	0.907 ± 0.012
**SpectralTCN** (A1 + WGM + GeM)	6.47	**0.225** ± **0.032**	**0.934** ± **0.004**	**0.922 ± 0.015**	**0.917** ± **0.017**

The ablation variants in Table 10 provide a progressive component-isolation analysis. A1 is the ModernTCN backbone without spectral processing. A2 adds FFT-based global spectral gating, while A3 replaces FFT with the proposed DWT-based WGM to assess the benefit of multi-resolution, time-localised filtering. A4 isolates the effect of GeM pooling without WGM. The full SpectralTCN combines WGM and GeM to evaluate their complementary contribution.

Replacing the FFT gate with the proposed WGM (A3) improves the test accuracy from 0.914 ± 0.018 to 0.917 ± 0.017 and reduces the validation loss from 0.247 to 0.227, suggesting that DWT-based time-localised filtering provides a small advantage over global FFT gating on this dataset. Although the direct gain over FFT is modest, the lower validation loss suggests more stable optimisation. GeM pooling alone (A4) also improves the backbone performance, achieving 0.913 ± 0.012 accuracy. The full SpectralTCN, which combines WGM and GeM, achieves the best overall result with 0.922 ± 0.015 test accuracy and the most stable validation accuracy of 0.934 ± 0.004. This suggests that WGM and GeM provide complementary benefits: WGM extracts discriminative sub-band features, while GeM adaptively emphasises their most informative temporal positions.

We also examined the effect of the residual-scalar initialisation in the WGM. Under the same training protocol, α = 0, 0.1, and 1.0 achieved test accuracies of 88.37%, 88.25%, and 90.40%, respectively. These are single-run values from a separate diagnostic set of runs and are therefore not directly comparable with Table 7. They indicate that SpectralTCN remains stable under different α initialisations; α = 1.0 gave the highest accuracy among the tested settings, but the spread of about 2 percentage points is comparable to the fold-to-fold variation reported in Table 10. We retain α = 0 because it preserves the identity mapping at initialisation and gave more stable early training.

To further examine which wavelet sub-bands contribute most to the performance of the proposed WGM, we performed a sub-band masking ablation on the Z24 Bridge test dataset as shown in Table 11. The trained db2 WGM was kept fixed, and each sub-band was individually masked during evaluation. This sub-band masking study is a separate diagnostic experiment; thus, its absolute scores should be interpreted relative to its own Full WGM baseline rather than directly compared with the preceding architectural ablation table. The largest F1 drops occur when a3 and d1 are removed, indicating that the global low-frequency structural response and the higher modal or transient-sensitive band carry the most damage-sensitive information, while d2 and d3 provide complementary contributions.

**Table 11:** Sub-band masking ablation of the db2 WGM on the Z24 dataset.

WGM setting	Effective Stage-1 band	Physical interpretation	Accuracy (%)	F1-macro (%)	F1 drop (%)
Full WGM	all bands	Adaptive multi-resolution filtering	**89.39 ± 0.90**	**89.39 ± 0.88**	0.00
w/o d1	3.125–6.25 Hz	Higher modal/ transient-sensitive band removed	81.41 ± 3.35	81.44 ± 3.36	7.95
w/o d2	1.5625–3.125 Hz	Mid-frequency modal band removed	87.91 ± 1.45	87.95 ± 1.39	1.44
w/o d3	0.781–1.562 Hz	Low-frequency detail band removed	88.97 ± 1.29	88.96 ± 1.28	0.43
w/o a3	0–0.781 Hz	Global low-frequency structural response removed	81.02 ± 5.22	80.69 ± 5.53	8.70

**Fig 12:**
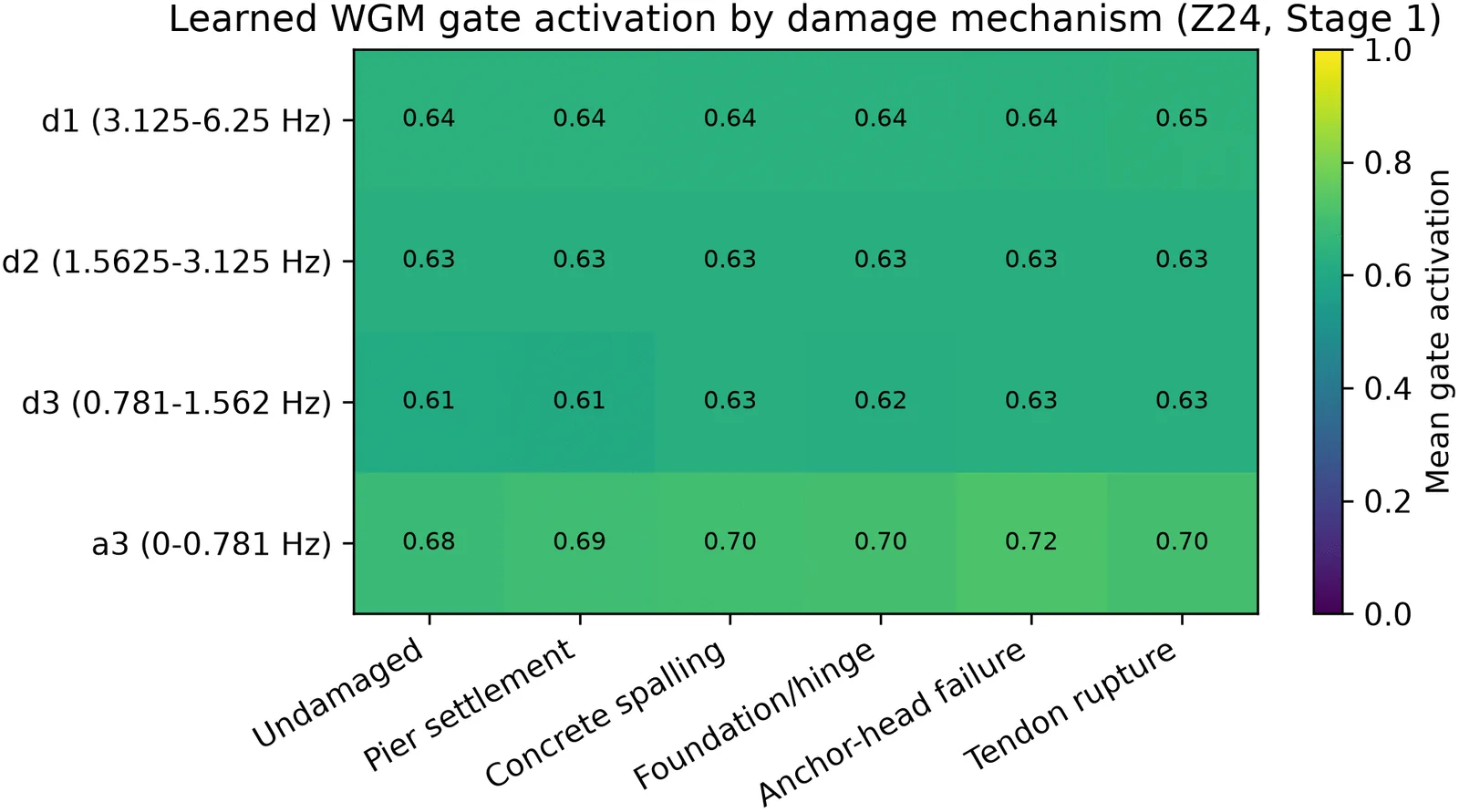
Mean WGM gate activation by damage mechanism on the Z24 Bridge (Stage 1), pooled over all 5 test folds.

To further verify that the learned WGM gates behave in an input-adaptive and reproducible manner rather than as a fixed filter, we visualised the mean gate activation per damage mechanism (Fig 12) and assessed its stability across the five independently trained cross-validation folds.

**Fig 13:**
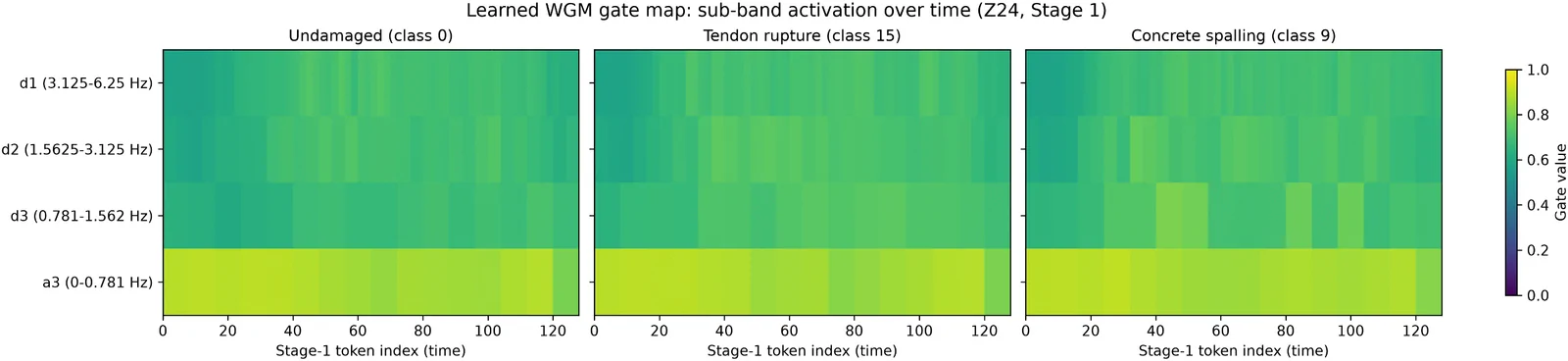
Learned WGM gate maps for representative Z24 windows.

The a3 sub-band (global low-frequency response, 0–0.781 Hz) shows a consistent increase in mean gate activation from the undamaged baseline (0.68) to damaged states (0.69–0.72, peaking at anchor-head failure). This shift is small relative to the cross-fold variability of a3 reported in Table 12 and should be read as a trend rather than a precise effect size. It is consistent with a3 being identified as the single most damage-sensitive band in the sub-band masking ablation above. The detail bands d1–d3 remain comparatively stable across damage mechanisms, indicating that the WGM concentrates its damage-adaptive modulation mainly in the low-frequency band while keeping the finer detail bands broadly open. Fig 13 shows the learned gate maps of individual Z24 windows. Within a single window, the gate value fluctuates over time rather than acting as a constant per-sample scalar (within-window temporal standard deviation of 0.02–0.05 across sub-bands), indicating that the gating is time-varying and input-dependent rather than a fixed, class-agnostic filter.

**Table 12:** Cross-fold stability of learned WGM gate activations (mean ± std across 5 independently trained folds, Stage 1, Z24 test sets).

Sub-band	Hz range (Stage 1)	Mean gate (5-fold)	Std	Coefficient of variation (%)
d1	3.125–6.25 Hz	0.642	0.053	8.2
d2	1.5625–3.125 Hz	0.628	0.057	9.0
d3	0.781–1.562 Hz	0.618	0.048	7.7
a3	0–0.781 Hz	0.698	0.089	12.8

The mean gate activation is consistent across the five independently trained folds, with coefficients of variation between 7.7% and 12.8% for all four sub-bands, indicating that the input-adaptive gating strategy learned by the WGM is stable and reproducible rather than an artefact of a single training run.

To justify the selection of the db2 wavelet and examine the influence of other important SpectralTCN hyperparameters, we conducted a one-factor-at-a-time sensitivity analysis on the Z24 Bridge dataset using the same 5-fold cross-validation splits. The wavelet-family comparison is reported in Table 13, and the remaining hyperparameter sweeps in Table 14.

**Table 13:** Sensitivity to wavelet family (Z24 Bridge, 5-fold CV, mean ± std; ROC-AUC macro reported as mean only).

Wavelet	Accuracy (%)	Balanced accuracy (%)	F1-macro (%)	MCC (%)	Cohen’s kappa (%)	ROC-AUC macro (%)
Haar	88.94 ± 1.14	88.94 ± 1.14	88.96 ± 1.09	88.27 ± 1.20	88.25 ± 1.21	99.57
**db2**	**90.03**± **1.28**	**90.03**± **1.28**	**90.07**± **1.28**	**89.42**± **1.36**	**89.40** ± **1.36**	**99.63**
db4	89.67 ± 0.66	89.67 ± 0.66	89.66 ± 0.64	89.04 ± 0.70	89.03 ± 0.70	99.60
sym4	89.54± 1.63	89.54± 1.63	89.54± 1.64	88.91± 1.72	88.89 ± 1.73	99.60

**Table 14:** Sensitivity to decomposition depth, kernel size, and GeM exponent.

Hyperparameter	Tested setting	F1-macro (%)
Default setting	db2, L = 3, k = 31, GeM p = 3	90.07 ± 1.28
Decomposition depth	L = 1/ 2/ 3/ 4	89.13/ 89.41/ **90.07**/ 89.73
Kernel size	k = 7/ 31	**93.18**/ 90.07
GeM exponent	p = 1/ 3/ 6	**92.25**/ 90.07/ 89.37

Unless noted otherwise, all values in Table 14 are reported on the Z24 Bridge dataset. The default-setting row (db2, L = 3, k = 31, GeM p = 3) corresponds to the same 5-fold cross-validation mean already reported for db2 in Table 13. The decomposition-depth, kernel-size, and GeM-exponent sweep results are single-run point estimates on the same Z24 split, evaluated without 5-fold averaging given the added computational cost of a full cross-validated sweep over every setting; they should therefore be interpreted as indicative of relative trends rather than as precise performance estimates. As with the sub-band masking study in Table 11, these sensitivity runs form a separate set of experiments; their absolute scores are therefore comparable within Tables 13 and 14, but not directly with the main cross-validation results in Table 7.

As shown in Tables 13 and 14, db2 achieves the highest mean performance among the tested wavelet families, supporting its use as the default wavelet basis in WGM. The model is also relatively stable with respect to decomposition depth, with L = 3 giving the best F1-macro among the tested levels. Kernel size and GeM exponent have a stronger influence, with k = 7 and GeM p = 1 achieving higher F1-macro on the Z24 dataset. However, the default configuration (k = 31 and GeM p = 3) was fixed before the main comparison experiments and used consistently across both datasets to avoid post-hoc tuning bias. Therefore, these sensitivity results are reported only to assess hyperparameter influence, rather than to re-select the final model after observing the test performance. Overall, SpectralTCN remains robust under different wavelet settings and may be further improved through task-specific hyperparameter tuning.

#### Qualitative analysis.

**Fig 14:**
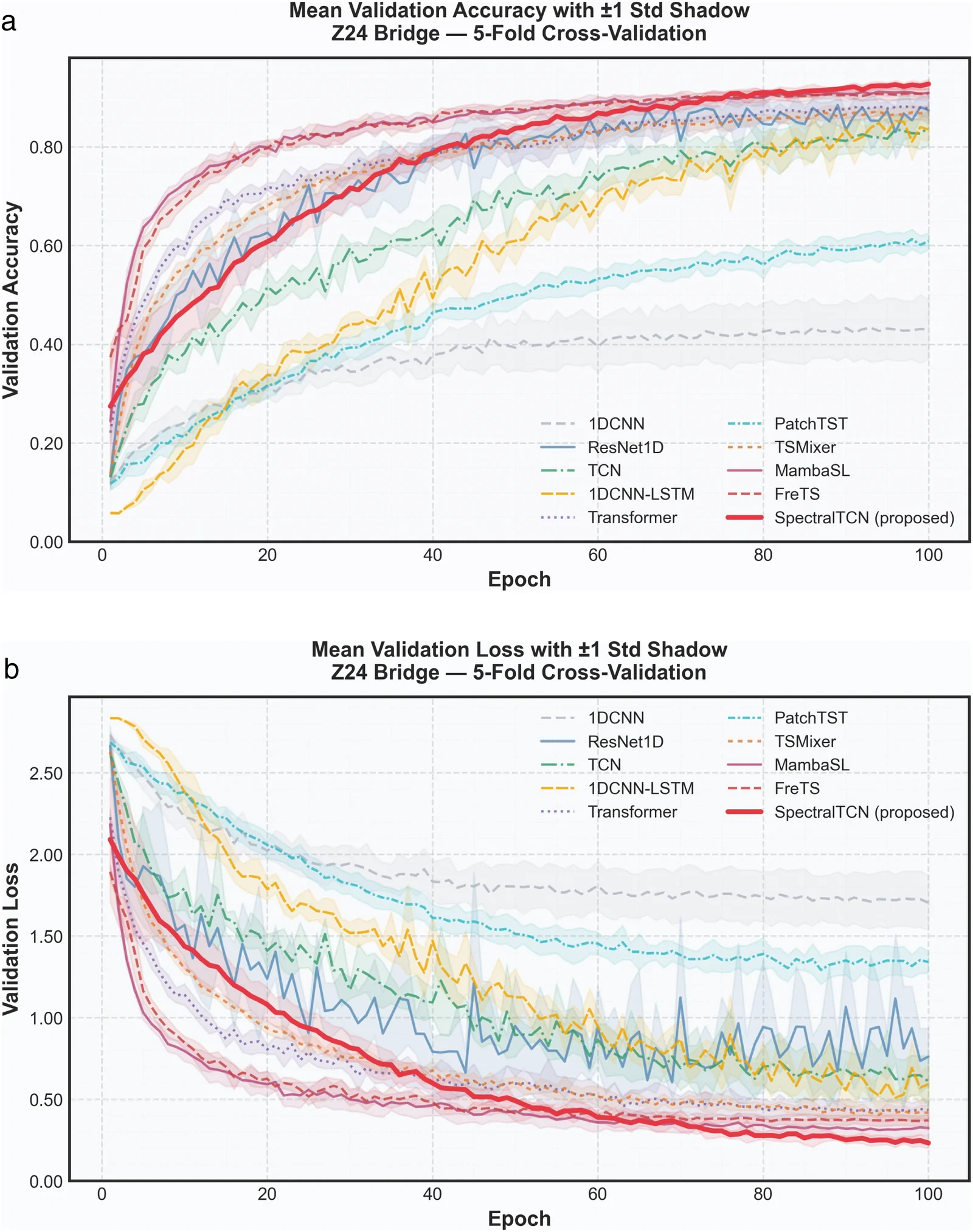
Mean validation accuracy and loss with ±1 standard deviation shadow across 5-fold cross-validation for all baseline models and SpectralTCN on the Z24 Bridge. (a) Validation accuracy; (b) validation loss.

The learning curves in Fig 14 corroborate the quantitative results: SpectralTCN and ResNet1D converge to the highest validation accuracy, while lighter models such as 1DCNN and PatchTST plateau at substantially lower accuracy levels from early epochs.

**Fig 15:**
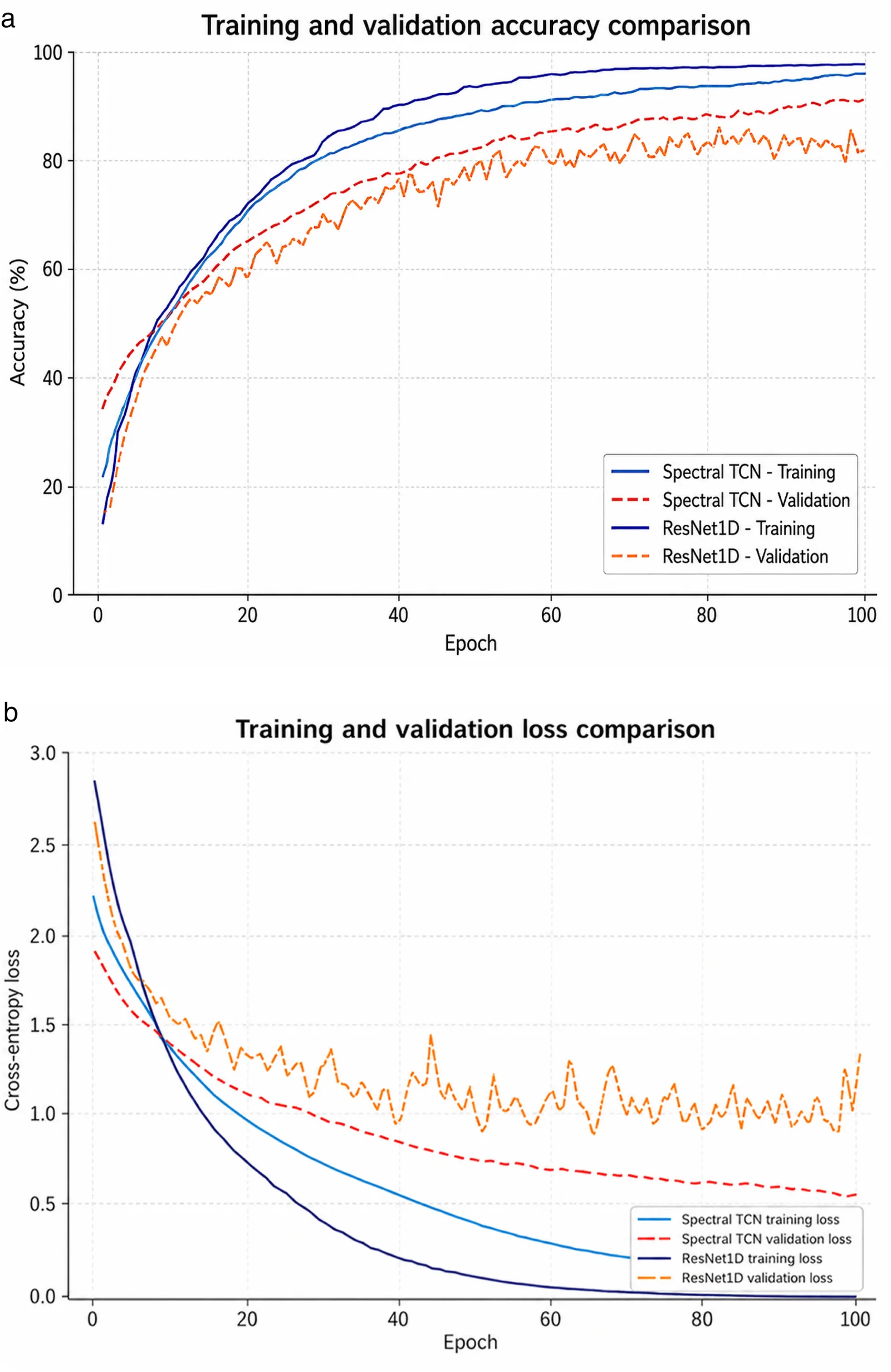
Five-fold averaged training and validation accuracy/loss histories of SpectralTCN and ResNet1D on the Z24 Bridge dataset. (a) Accuracy; (b) loss.

To further examine the possibility of overfitting, Fig 15 shows the training and validation accuracy/loss histories of SpectralTCN and ResNet1D, the two best-performing models on the Z24 dataset. For both models, validation accuracy follows training accuracy closely, and validation loss remains stable without clear divergence, indicating stable convergence. Compared with ResNet1D, SpectralTCN shows a smaller and more stable generalisation gap, while ResNet1D exhibits stronger validation-loss fluctuations. These trends suggest that SpectralTCN is less prone to overfitting and generalises more robustly under the current dataset size.

**Fig 16:**
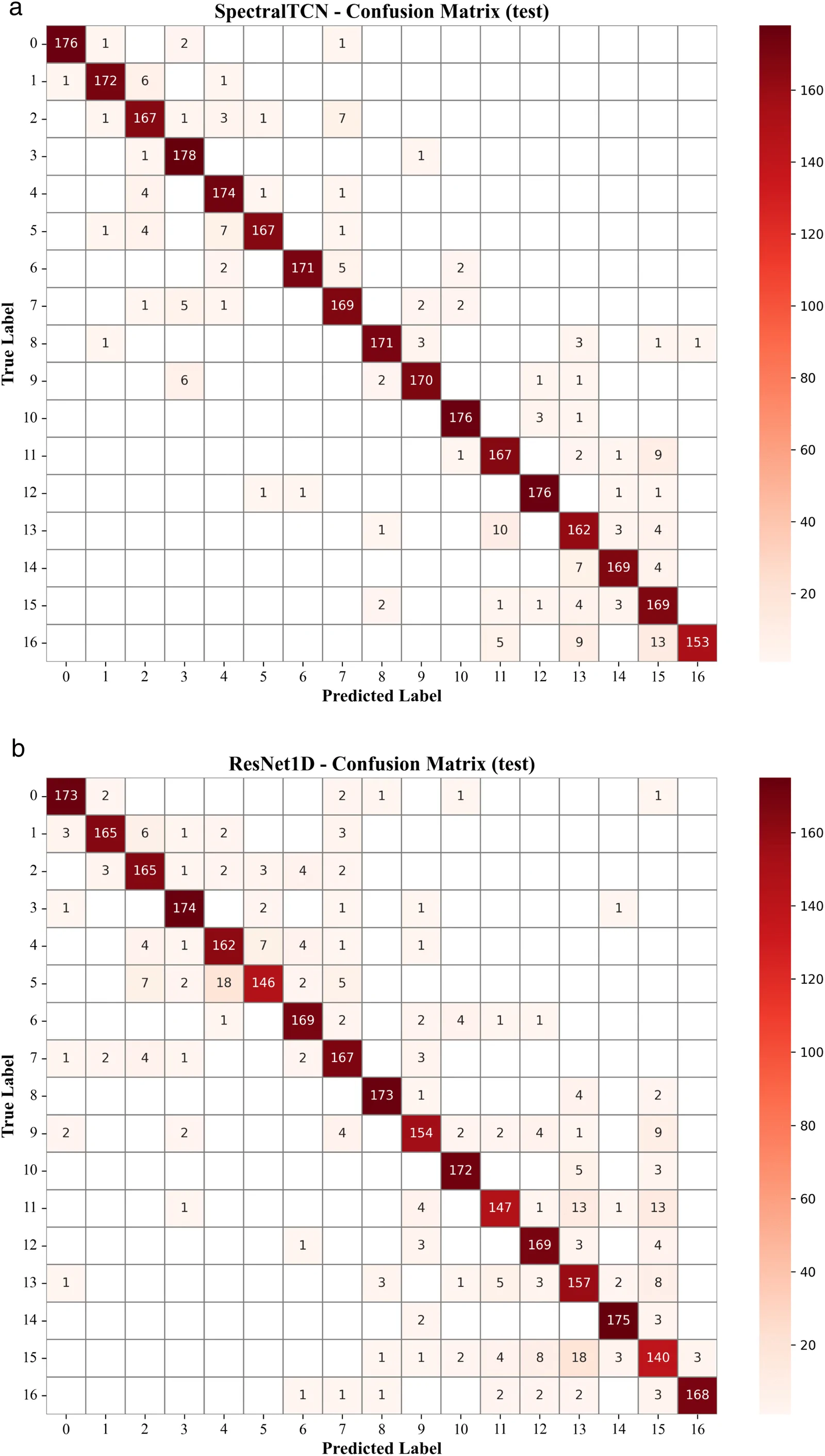
Normalised confusion matrices on the Z24 Bridge test set (fold 4) for SpectralTCN and ResNet1D. (a) SpectralTCN; (b) ResNet1D.

The normalised confusion matrices in Fig 16 provide a per-class view of the classification errors on fold 4. SpectralTCN achieves near-diagonal structure across all 17 structural states, with the largest off-diagonal entries at States 2, 11, 13 and 15. These correspond to a small pier settlement (20 mm), concrete-hinge failure, and anchorage/tendon damage – mechanisms that alter the global stiffness only marginally and therefore produce closely overlapping modal signatures. ResNet1D shows broader scatter, particularly at States 4, 5, 7 and 13. Notably, it confuses State 7 – the second reference (undamaged) condition – with the adjacent pier-settlement states, indicating that it does not reliably separate the healthy baseline from small stiffness reductions, whereas SpectralTCN keeps State 7 at F1 = 0.929 (Table 15).

**Table 15:** Per-class precision (P), recall (R), and F1-score of SpectralTCN and ResNet1D on the Z24 Bridge test set (fold 4).

Damage state	SpectralTCN			ResNet1D		
	P	R	F1	P	R	F1
State 0	0.9944	0.9778	0.9860	0.9058	0.9611	0.9326
State 1	0.9773	0.9556	0.9663	0.9713	0.9389	0.9548
State 2	0.9126	0.9278	0.9201	0.9270	0.9167	0.9218
State 3	0.9271	0.9889	0.9570	0.9351	0.9611	0.9479
State 4	0.9255	0.9667	0.9457	0.9167	0.7944	0.8512
State 5	0.9824	0.9278	0.9543	0.7951	0.9056	0.8468
State 6	0.9942	0.9500	0.9716	0.9529	0.9000	0.9257
State 7	0.9185	0.9389	0.9286	0.8256	0.8944	0.8587
State 8	0.9716	0.9500	0.9607	0.9301	0.9611	0.9454
State 9	0.9659	0.9444	0.9551	0.9620	0.8444	0.8994
State 10	0.9724	0.9778	0.9751	0.9581	0.8889	0.9222
State 11	0.9126	0.9278	0.9201	0.8063	0.8556	0.8302
State 12	0.9724	0.9778	0.9751	0.8994	0.8444	0.8711
State 13	0.8571	0.9000	0.8780	0.7610	0.8667	0.8104
State 14	0.9548	0.9389	0.9468	0.9451	0.9556	0.9503
State 15	0.8408	0.9389	0.8871	0.8779	0.8389	0.8580
State 16	0.9935	0.8500	0.9162	0.9318	0.9111	0.9213
Accuracy	**0.9435**			0.8964		
MCC	**0.9400**			0.8901		

**Fig 17:**
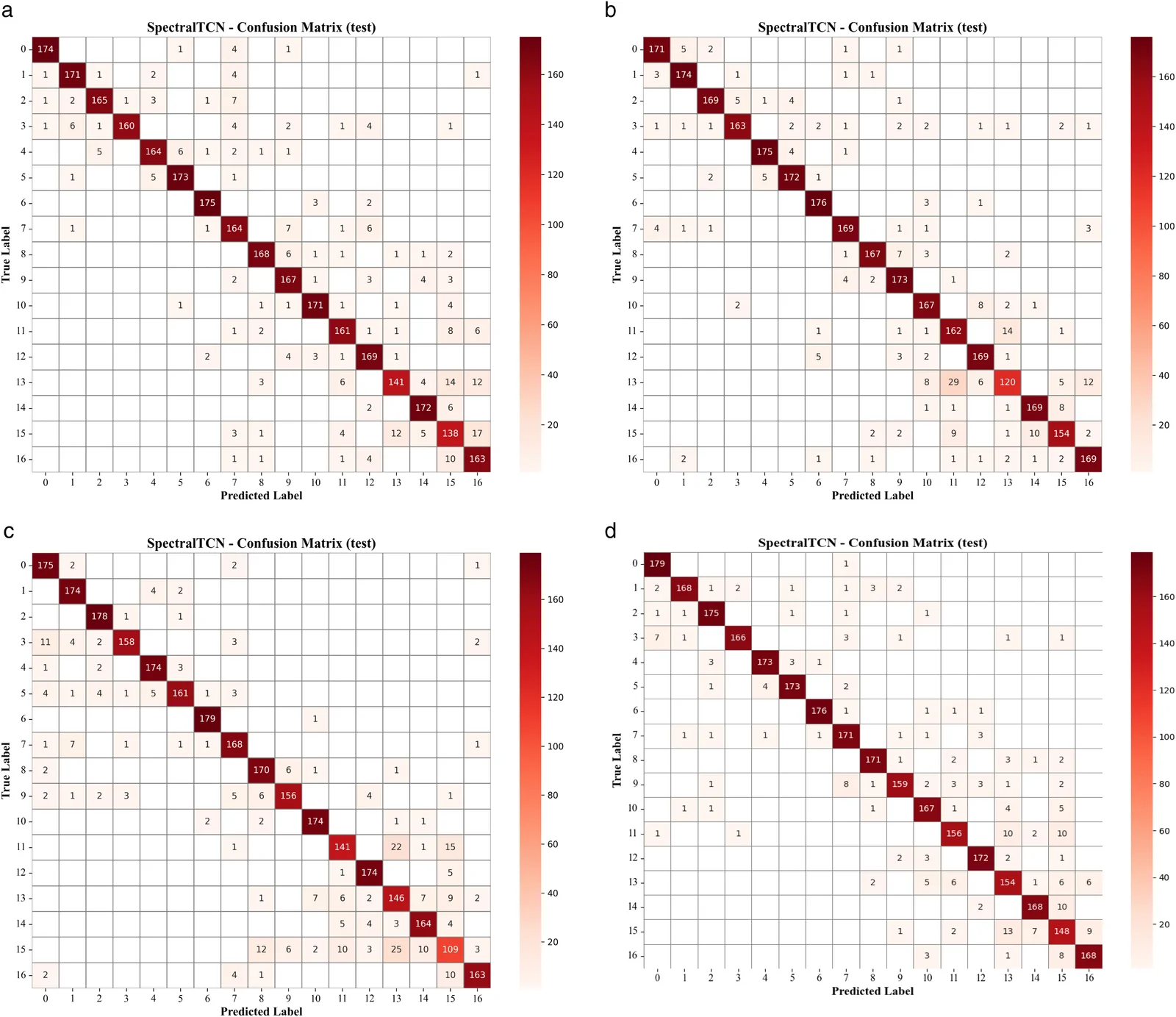
Normalised confusion matrices of SpectralTCN on the Z24 Bridge test sets for the remaining folds. (a) Fold 0; (b) fold 1; (c) fold 2; (d) fold 3.

Additional fold-wise confusion matrices in Fig 17 show that SpectralTCN maintains predominantly diagonal prediction patterns across folds.

**Fig 18:**
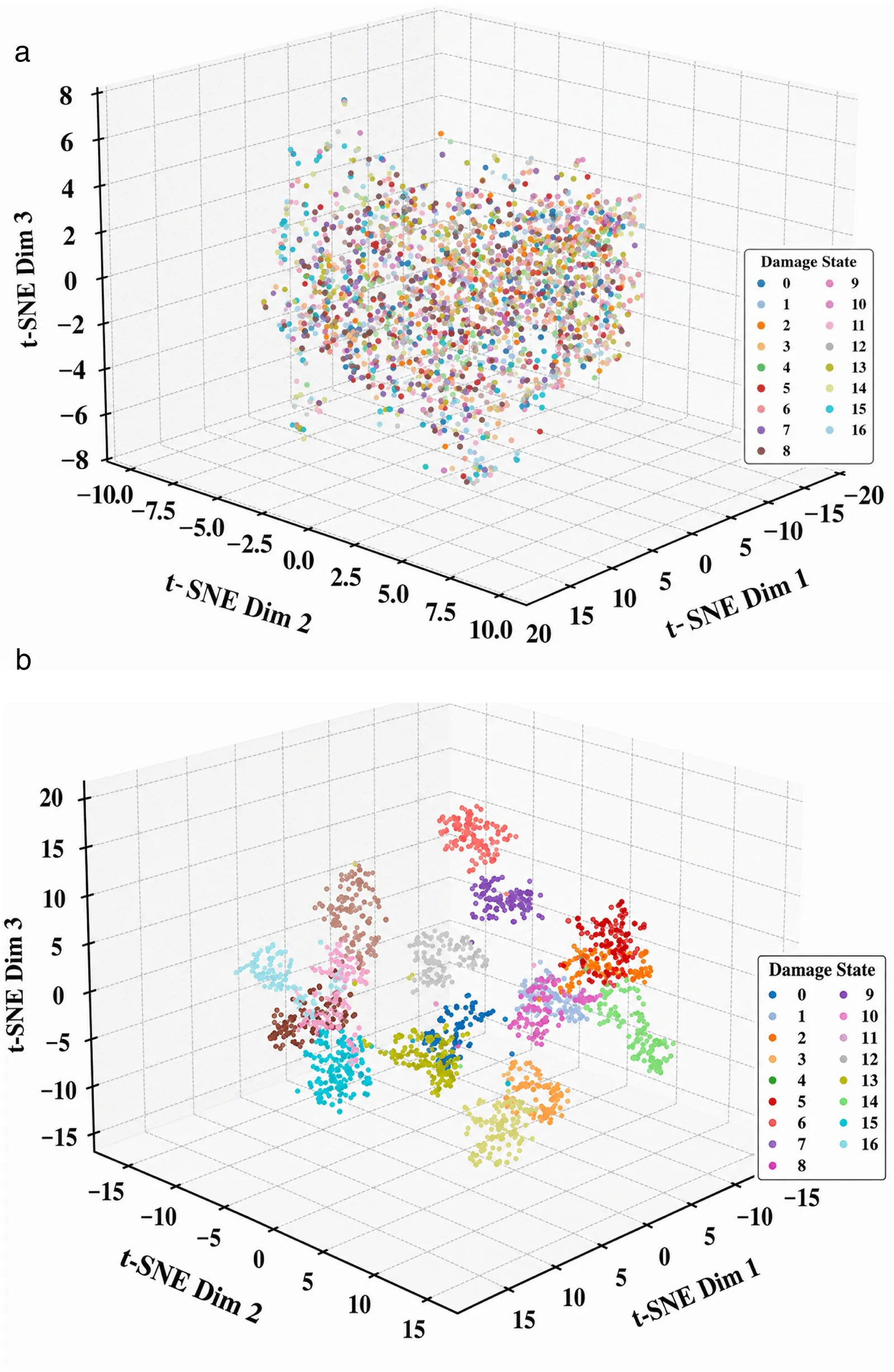
3D t-SNE visualisation of SpectralTCN penultimate-layer features on the Z24 Bridge training set at epoch 1 (a) and the final training epoch (b).

Fig 18 shows the 3D t-SNE projection of SpectralTCN penultimate-layer features on the Z24 training set at two training snapshots. At epoch 1, the 17 structural states are heavily overlapping, reflecting the randomly initialised network. By the final epoch, the classes form largely separated clusters in the embedding space; because the projection is computed on the training set, it illustrates what the network has fitted rather than how well it generalises.

Table 15 details the per-class precision, recall, and F1-score for SpectralTCN and ResNet1D on Z24 fold 4. SpectralTCN achieves F1-scores above 0.87 on all 17 structural states, with the lowest values at States 13 (F1 = 0.878), 15 (0.887), and 16 (0.916), which correspond to anchor-head failures and tendon ruptures that produce subtle, low-amplitude changes in the higher natural frequencies. ResNet1D shows more pronounced drops at States 4, 5, 7, and 13, confirming the qualitative observation from the confusion matrices that ResNet1D struggles to distinguish spectrally similar progressive-damage states without the multi-resolution filtering provided by the WGM.

### Results on My Thuan Bridge

#### Comparison with baseline models.

Table 16 reports the classification performance on the My Thuan Bridge FEM dataset. SpectralTCN achieves the highest accuracy of 0.921 ± 0.020, outperforming the second-best model, Transformer (0.889± 0.011), by 3.2 percentage points. Attention-based and state-space models perform notably better on this dataset than purely convolutional baselines: Transformer (0.889), MambaSL (0.873 ±0.032), and TSMixer (0.865±0.031) all exceed 0.86, while conventional temporal models such as 1DCNN (0.664), FreTS (0.662), TCN (0.725), and 1DCNN-LSTM (0.747) are substantially lower. This pattern suggests that the FEM-generated vibration signals contain longer-range temporal dependencies that benefit global context modelling, but the full multi-resolution spectral processing of SpectralTCN provides an additional advantage over global attention alone. PatchTST achieves 0.549±0.190, with high fold-to-fold variance (fold 0 collapses to 0.179 while fold 3 reaches 0.715), suggesting that the patch-embedding design is sensitive to initialisation on this smaller dataset. SpectralTCN’s WGM enables data-dependent per-band filtering aligned with the bridge’s structural vibration modes, providing the performance margin over the Transformer by simultaneously preserving temporal localisation and physically interpretable frequency selectivity. Table 16 also includes GRU as a lightweight baseline, together with Param (M) and MACs/window for neural-network-based models. The results show that SpectralTCN obtains the best overall classification performance at 153.44 M MACs per window, below PatchTST (1807.15 M), TSMixer (226.49 M) and TCN (210.70 M), although its 6.47 M parameters make it the largest model in the comparison. SpectralTCN therefore offers a favourable accuracy-versus-MACs trade-off, at the cost of a larger memory footprint and, as shown in Table 17, a longer per-window inference time.

**Table 16:** Classification performance on the My Thuan Bridge (5-fold CV, mean ± std, 11 classes). Best in bold, second best underlined.

Model	Param (M)	Accuracy	F1-macro	MCC	MACs/window (M)
1DCNN	0.02	0.664 ± 0.095	0.629 ±0.107	0.651 ± 0.097	5.57
ResNet1D	0.03	0.759 ± 0.074	0.754 ± 0.076	0.737 ±0.080	25.53
TCN	0.20	0.725 ± 0.064	0.707 ± 0.071	0.703 ± 0.070	210.70
1DCNN-LSTM	0.72	0.747 ± 0.058	0.741 ± 0.064	0.723 ± 0.062	205.26
Transformer	0.87	0.889 ± 0.011	0.887 ± 0.012	0.878 ± 0.012	38.27
PatchTST	0.61	0.549 ±0.190	0.543 ± 0.190	0.508 ± 0.206	**1807.15**
TSMixer	4.28	0.865 ± 0.031	0.856 ± 0.031	0.854 ±0.034	226.49
MambaSL	0.19	0.873 ± 0.032	0.872 ± 0.033	0.861 ± 0.035	23.14
FreTS	0.28	0.662 ± 0.066	0.649 ± 0.075	0.633 ± 0.071	44.92
GRU	0.43	0.723 ± 0.229	0.722 ± 0.230	0.702 ± 0.240	107.74
**SpectralTCN**	**6.47**	**0.921** ± **0.020**	**0.920** ± **0.020**	**0.914** ± **0.022**	153.44

**Table 17:** Computational cost comparison between TCN, ModernTCN without WGM, and SpectralTCN on the My Thuan Bridge dataset (single input window).

Model	Param (M)	MACs/window (M)	FLOPs/window (M)	Inference time (ms)	Throughput (windows/s)
TCN	0.20	210.70	421.41	2.587	386.6
ModernTCN w/o WGM	4.63	118.26	236.53	5.030	198.8
SpectralTCN	6.47	153.44	306.88	24.023	41.6

To further complement the parameter and MAC comparisons reported in Tables 7 and 16, Table 17 provides a focused computational-cost comparison of TCN, ModernTCN without WGM, and SpectralTCN in terms of parameters, MACs, FLOPs, inference time, and throughput under the same inference setting for one input window.

As shown in Table 17, the WGM increases the inference time of SpectralTCN compared with ModernTCN without WGM, mainly due to the DWT/IDWT and sub-band gating operations; however, this additional cost is accompanied by the best classification performance on both datasets.

**Fig 19:**
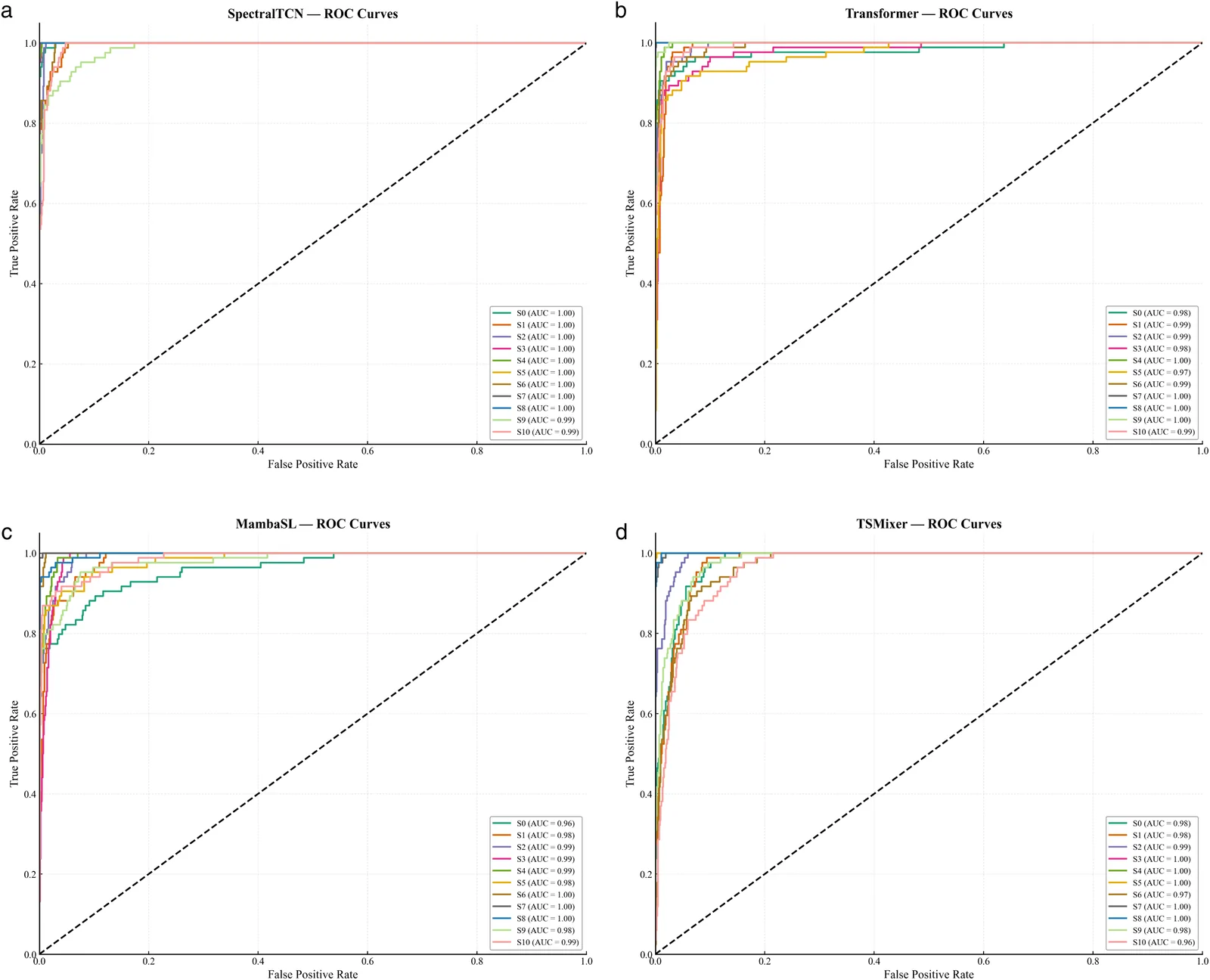
Macro-averaged ROC-AUC curves on the My Thuan Bridge for the four best-performing models in fold 4. Each curve corresponds to one of the 11 structural states. (a) SpectralTCN; (b) Transformer; (c) MambaSL; (d) TSMixer.

The ROC-AUC curves in Fig 19 illustrate the per-class discrimination quality of the four best models on the My Thuan dataset. SpectralTCN achieves consistently high one-versus-rest separation across all 11 structural states, while the Transformer, MambaSL, and TSMixer show slightly wider variation particularly for states with similar frequency signatures.

#### Ablation study.

Table 18 presents the ablation results on the My Thuan dataset. The ModernTCN backbone (A1) achieves 0.831 ±0.069 test accuracy, indicating that the base architecture already performs reasonably on the FEM dataset. Adding FFT-based spectral gating (A2) yields only marginal change (0.825 ±0.078), while adding WGM alone (A3, 0.798 ± 0.082) and GeM alone (A4, 0.793 ± 0.078) both underperform the backbone, by 3.3 and 3.8 percentage points respectively. The validation losses for A3 (1.118 ± 0.674) and A2 (0.809 ± 0.417) are notably higher and more variable than A1 (0.532 ± 0.227), suggesting that individual spectral components introduce optimisation difficulty when deployed in isolation on FEM-generated data. The full SpectralTCN (WGM + GeM combined) achieves a clear improvement to 0.921 ± 0.020 with a validation loss of 0.314 ± 0.131 – the lowest among all variants and 41% below that of the backbone (0.532 ± 0.227) – demonstrating that WGM and GeM operate synergistically: multi-resolution frequency gating reduces spectral noise while GeM pooling aggregates the resulting features with appropriate emphasis on discriminative temporal positions.

**Table 18:** Ablation study on the My Thuan Bridge (5-fold CV, mean ± std). Best result in bold.

Variant	Param(M)	Val Loss	Val Acc	Test Acc	MCC
A1: ModernTCN backbone	4.63	0.532 ± 0.227	0.878± 0.031	0.831± 0.069	0.816 ± 0.075
A2: A1 + FFT	5.17	0.809 ± 0.417	0.858± 0.036	0.825± 0.078	0.809 ± 0.086
A3: A1 + WGM	5.71	1.118 ± 0.674	0.774± 0.123	0.798± 0.082	0.780± 0.090
A4: A1 + GeM	4.63	0.646± 0.260	0.851± 0.037	0.793± 0.078	0.774± 0.084
**SpectralTCN (A1 + WGM + GeM)**	**6.47**	**0.314** ± **0.131**	**0.912** ± **0.032**	**0.921** ± **0.020**	**0.914** ± **0.022**

The My Thuan ablation results further show that the proposed components are most effective when combined rather than used in isolation. While FFT, WGM, or GeM alone do not consistently improve the ModernTCN backbone on this FEM-generated dataset, the full SpectralTCN substantially improves both test accuracy and validation loss. This suggests that WGM provides adaptive multi-resolution filtering, whereas GeM aggregates the resulting damage-sensitive temporal features more effectively. Therefore, the full model benefits from the interaction between spectral filtering and learnable temporal pooling.

#### Qualitative analysis.

**Fig 20:**
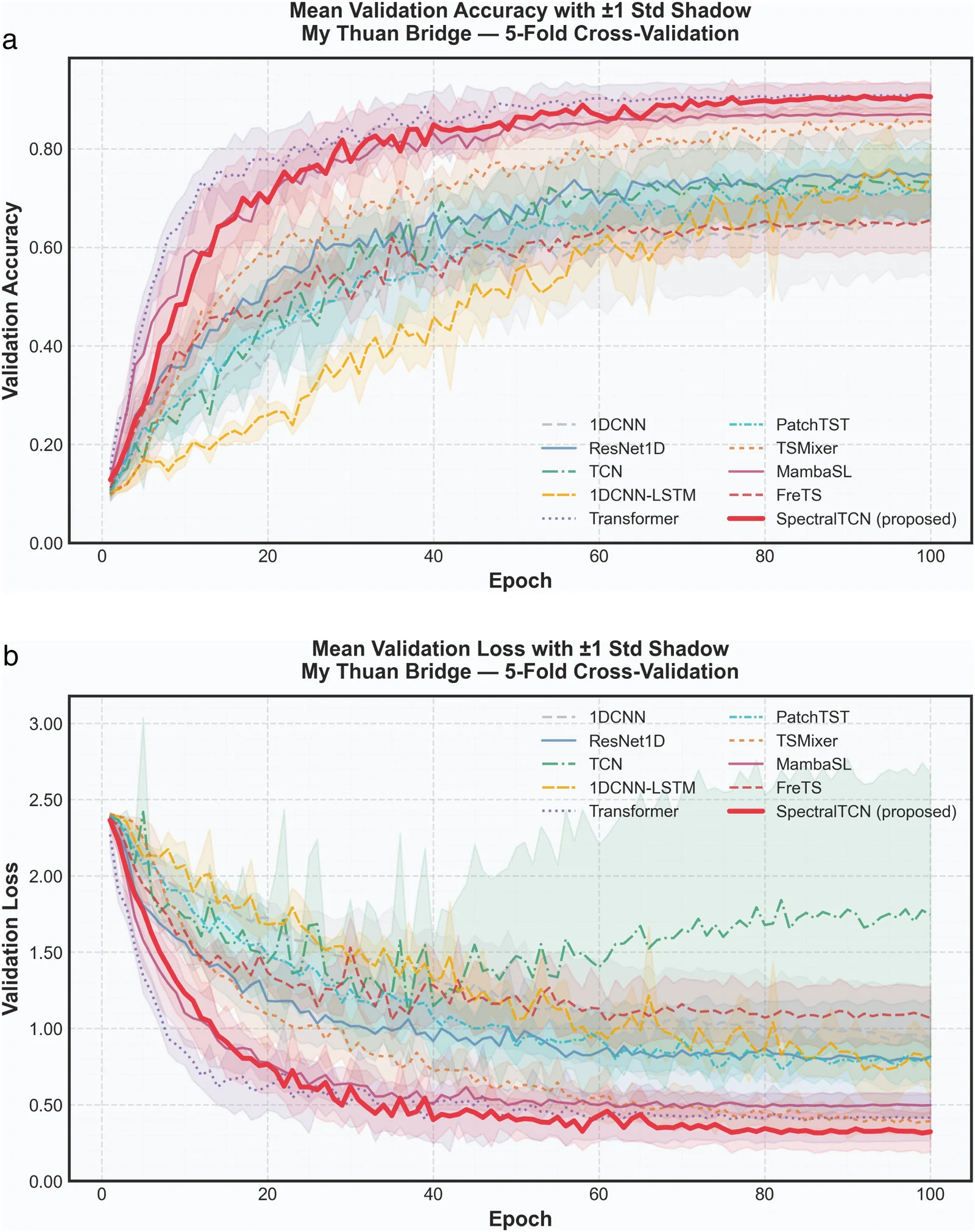
Mean validation accuracy and loss with ±1 standard deviation shadow across 5-fold cross-validation for all baseline models and SpectralTCN on the My Thuan Bridge. (a) Validation accuracy; (b) validation loss.

The learning curves in Fig 20 illustrate the instability of most baseline models on this dataset: several models exhibit wide shadow bands indicating large fold-to-fold variation, while SpectralTCN converges smoothly to a high and stable validation accuracy with a narrow shadow band.

**Fig 21:**
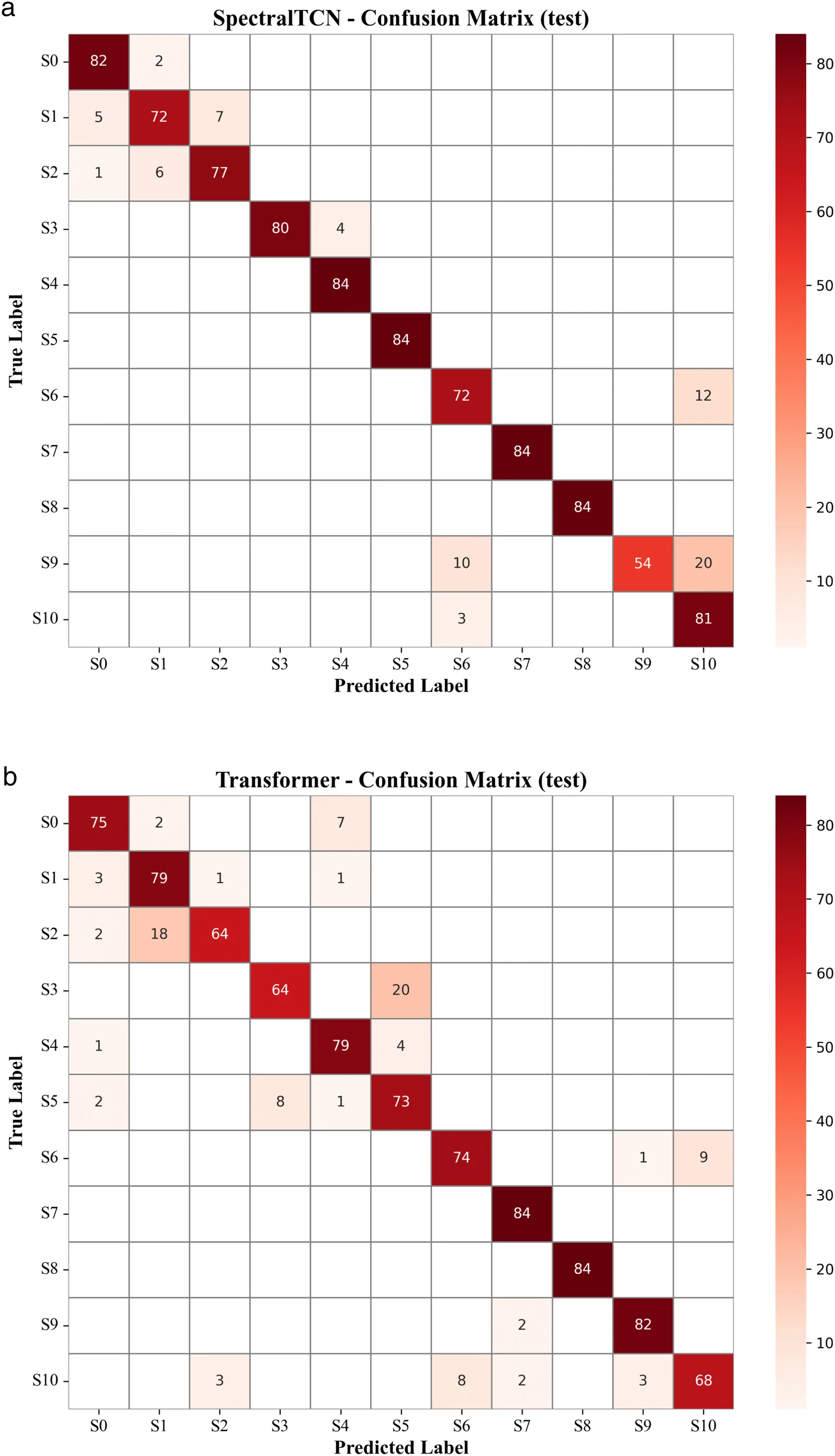
Normalised confusion matrices on the My Thuan Bridge test set (fold 4) for SpectralTCN and Transformer. Rows are true labels; columns are predicted labels; diagonal values indicate per-class recall. (a) SpectralTCN; (b) Transformer.

The confusion matrices in Fig 21 provide a per-class view of the classification errors. SpectralTCN achieves consistently high recall on most structural states, with the main confusions concentrated between S9 and S10, two damage scenarios involving similar stiffness reductions on adjacent members that produce overlapping modal frequency shifts. The Transformer exhibits wider off-diagonal scatter, particularly between S2–S3 and S3–S5, reflecting its tendency to confuse structurally similar states with comparable global frequency profiles when temporal localisation is not available.

**Fig 22:**
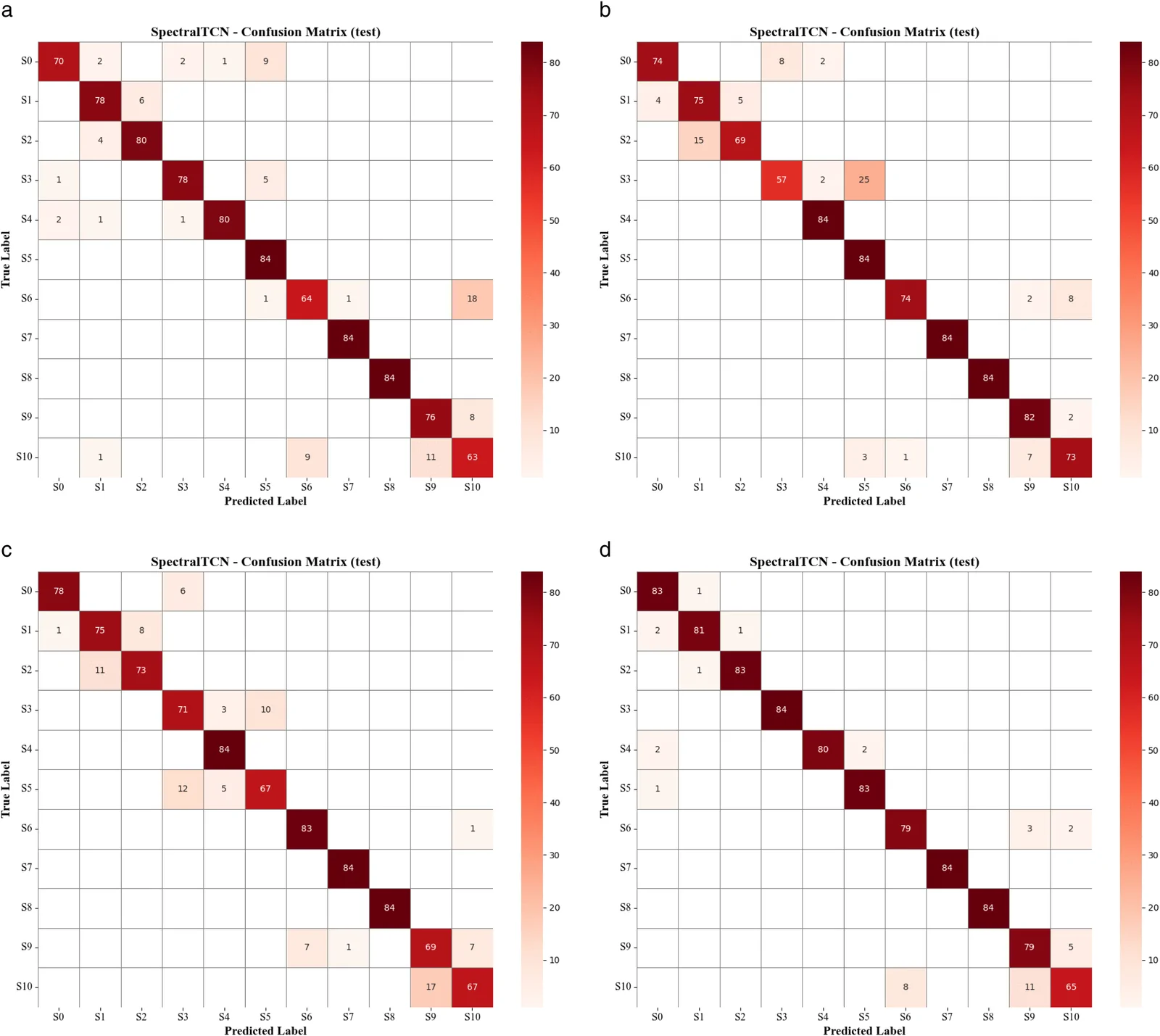
Normalised confusion matrices of SpectralTCN on the My Thuan Bridge test sets for the remaining folds. (a) Fold 0; (b) fold 1; (c) fold 2; (d) fold 3.

Fig 22 shows additional fold-wise confusion matrices, indicating that the class-wise prediction pattern is not specific to a single fold.

**Fig 23:**
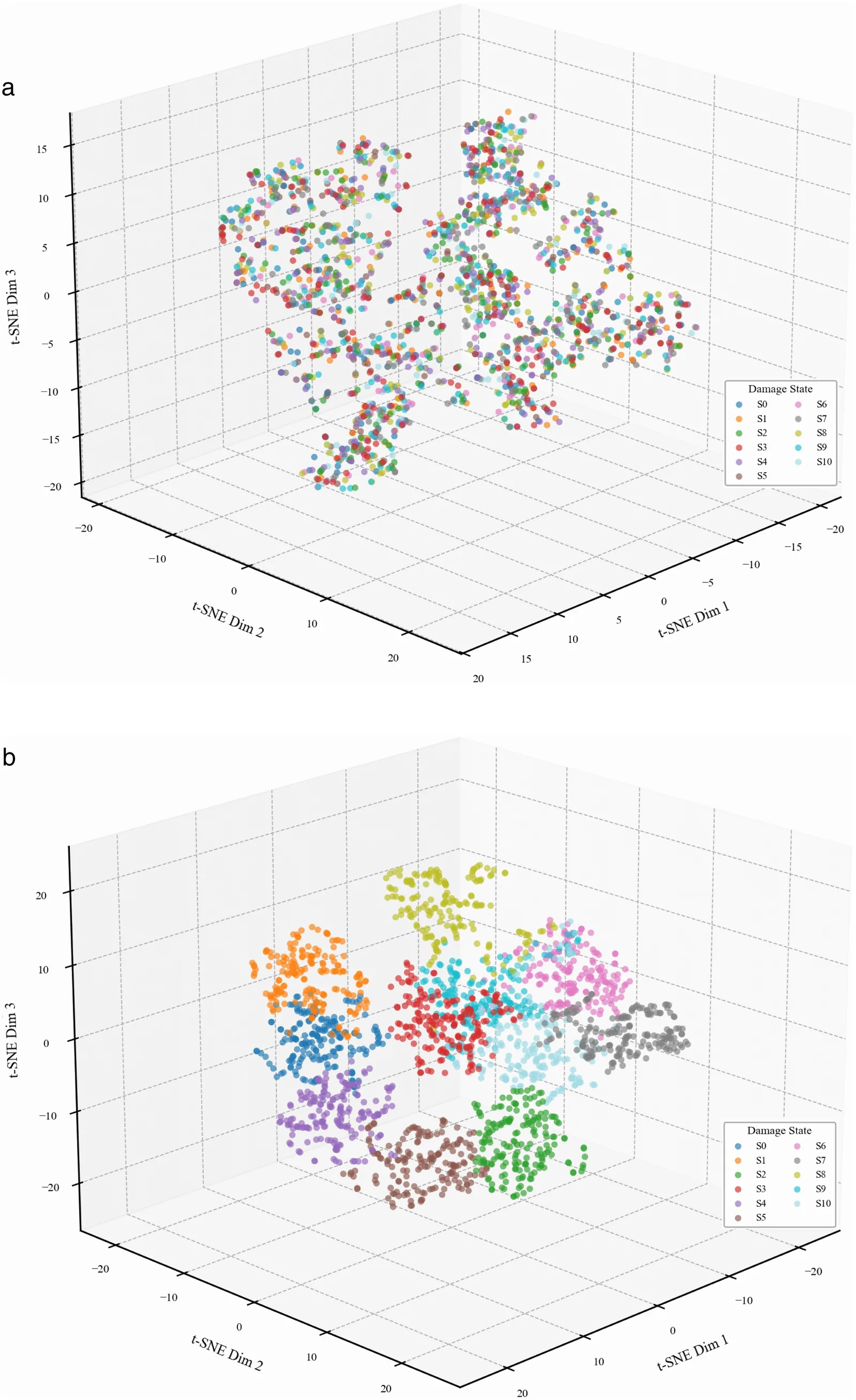
3D t-SNE visualisation of SpectralTCN penultimate-layer features on the My Thuan Bridge training set at epoch 1 (a) and the final training epoch (b).

Fig 23 presents the 3D t-SNE visualisation of SpectralTCN’s penultimate-layer features on the My Thuan dataset. At epoch 1, the 11 structural states are largely overlapping, reflecting the randomly initialised network. By the final epoch, distinct and well-separated clusters emerge for most structural states, suggesting that the learned representation becomes more discriminative after training even from the more challenging FEM-generated signals.

Table 19 reports per-class metrics for SpectralTCN and the second-best Transformer on fold 4. SpectralTCN achieves perfect F1 on three states (S5, S7, S8) and strong performance across the remaining classes, with the exception of S9 (recall 0.64) and S10 (precision 0.72), which involve structurally similar stiffness-reduction patterns that are challenging to distinguish. The Transformer obtains near-perfect recall on S1 (0.94) and S9 (0.98) but struggles with precision on S1 (0.80) and recall on S2 (0.76) and S3 (0.76), where its global attention mechanism confuses states with overlapping modal frequency shifts.

**Table 19:** Per-class precision (P), recall (R), and F1-score of SpectralTCN and Transformer on the My Thuan Bridge test set (fold 4).

State	SpectralTCN			Transformer		
	P	R	F1	P	R	F1
S0	0.9318	0.9762	0.9535	0.9036	0.8929	0.8982
S1	0.9000	0.8571	0.8780	0.7980	0.9405	0.8634
S2	0.9167	0.9167	0.9167	0.9412	0.7619	0.8421
S3	1.0000	0.9524	0.9756	0.8889	0.7619	0.8205
S4	0.9545	1.0000	0.9767	0.8977	0.9405	0.9186
S5	1.0000	1.0000	1.0000	0.7526	0.8690	0.8066
S6	0.8471	0.8571	0.8521	0.9024	0.8810	0.8916
S7	1.0000	1.0000	1.0000	0.9545	1.0000	0.9767
S8	1.0000	1.0000	1.0000	1.0000	1.0000	1.0000
S9	1.0000	0.6429	0.7826	0.9535	0.9762	0.9647
S10	0.7168	0.9643	0.8223	0.8831	0.8095	0.8447
Accuracy	**0.9242**			0.8939		
MCC	**0.9177**			0.8838		

### Cross-dataset discussion and engineering interpretation

Across both datasets, SpectralTCN achieves the best performance with low fold-to-fold variation, suggesting that the proposed components generalise beyond a single bridge type or data source. Although the real-world Z24 dataset and the FEM-derived My Thuan dataset differ in sensor layout, sampling rate, damage scenarios, and data-generation mechanism, both reflect damage-induced changes in vibration responses. In Z24, damage such as pier settlement, concrete spalling, anchorage damage, and tendon rupture affects stiffness distribution, boundary conditions, and load-transfer paths. In My Thuan, damage is simulated through Young’s modulus reductions in selected members, representing localised stiffness loss. Thus, both datasets are ultimately governed by stiffness-dependent modal changes.

This may help to explain the benefit of WGM’s multi-resolution spectral representation. Unlike global FFT-based gating, wavelet decomposition preserves both frequency-band information and temporal localisation, allowing the model to capture damage-sensitive features that may occur only during specific time intervals or excitation conditions. Through input-adaptive gating, WGM can emphasise sub-bands related to modal shifts and localised transient responses while suppressing operational variation, noise, and simulation-specific periodic components. Therefore, WGM is not only beneficial for classification accuracy, but also physically consistent with vibration-based damage mechanisms.

The baseline behaviour differs between the two datasets. On Z24, strong baselines such as ResNet1D, MambaSL, and FreTS also perform competitively, suggesting that the dataset is sufficiently large and structured for different architectures to train reliably. On My Thuan, attention-based and state-space models outperform conventional convolutional baselines, indicating longer-range temporal structure in the FEM-generated signals. Nevertheless, SpectralTCN remains the most stable model across both datasets, suggesting that explicit multi-resolution spectral processing is useful for both real monitoring data and simulation-derived responses. The WGM can suppress less informative bands while retaining the modal components that carry damage-related information.

More specifically, several properties of the My Thuan FEM benchmark make it a more challenging classification problem. Compared with Z24, it has lower intra-class variability because the responses are generated from a controlled numerical model and then segmented into windows, which can increase fold-to-fold variance. In addition, the simulated states share similar excitation and modelling assumptions, so damage information mainly appears as subtle shifts in modal-frequency components rather than clearly distinct signal patterns. Some damage cases also involve stiffness reductions in structurally similar members, causing overlapping modal and time-frequency signatures, especially among confused states such as S6, S9, and S10. These factors make My Thuan intrinsically harder than Z24 and highlight the benefit of WGM, whose input-adaptive multi-resolution gating can preserve temporal localisation and emphasise damage-sensitive frequency sub-bands.

Because the My Thuan dataset is FEM-generated, simulation-specific artefacts remain a possible limitation. However, three observations suggest that the model primarily exploits damage-related vibration features. First, the main confusions occur between structurally similar states such as S9 and S10. Second, damage-sensitive information is expected mainly in modal-frequency bands, whereas many numerical artefacts appear as less informative high-frequency components that can be attenuated by WGM. Third, the same architecture also performs strongly on the real-world Z24 dataset, where FEM-specific artefacts are absent. Nevertheless, we acknowledge that FEM data cannot be completely free of simulation-specific characteristics, which is why the method is validated on both the simulated My Thuan dataset and the real Z24 bridge recordings.

A further limitation concerns the frequency range covered by the WGM. Because the module operates on the patch-embedded sequence rather than on the raw acceleration signal, the Nyquist frequency of its sub-bands at Stage 1 is 6.25 Hz for Z24 and 31.25 Hz for My Thuan. The Z24 sub-bands therefore resolve the first two bending modes of the bridge explicitly, while higher modes are represented only implicitly through the learned stem embedding; this is consistent with the sub-band masking results in Table 11, where a3 and d1 dominate. Applying the WGM before the patch embedding, or with a smaller patch stride, would give direct access to higher modal bands at a higher computational cost and is left for future work.

## Conclusions

This paper proposes SpectralTCN, a ModernTCN-based temporal convolutional network enhanced with a WGM for vibration-based structural damage classification. The WGM introduces learnable, data-dependent multi-resolution filtering into the convolutional blocks using the DWT, allowing the network to adaptively amplify or suppress specific frequency sub-bands. Unlike FFT-based approaches, DWT preserves both time and frequency information, with decomposition levels corresponding to physically interpretable structural vibration bands. Together with GeM pooling and large-kernel causal depthwise convolutions, SpectralTCN achieves strong performance on two structurally distinct benchmarks.

Experiments against 10 baseline models under stratified 5-fold cross-validation show that SpectralTCN reaches 92.2% accuracy on the Z24 Bridge dataset and 92.1% accuracy on the My Thuan FEM dataset, achieving the strongest overall performance among the compared methods. The improvement over the strongest baseline is consistent across all five folds (Wilcoxon p = 0.031), although for accuracy, F1-macro and MCC the paired t-test does not reach the 5% significance level (p ≈ 0.07) given the limited number of folds; confirming the effect at a higher significance level would require repeated cross-validation over multiple random seeds. The ablation study confirms the contribution of the main components: (1) WGM provides the largest individual gain over the ModernTCN backbone on the Z24 dataset; (2) on the Z24 dataset, DWT-based multi-resolution gating provides a small benefit over FFT-based global spectral gating, although this benefit is not observed when the WGM is used in isolation on the My Thuan dataset; and (3) GeM pooling further improves temporal aggregation of localised damage features. Finally, because SpectralTCN is fully causal and processes short sliding windows with a purely convolutional backbone of moderate computational cost, it remains suitable for **near-real-time online abnormality detection** in continuous automated bridge monitoring.

The consistent performance across a real-world monitored bridge (Z24) and a simulated cable-stayed bridge (My Thuan) suggests that the proposed multi-resolution wavelet gating strategy generalises across different sensor configurations, and data sources. Future work will explore (1) learnable wavelet filter banks to replace the fixed db2 filters, (2) extension to online monitoring scenarios and multi-modal sensor configurations, and (3) regression-based extensions replacing the softmax classifier with a linear head for continuous damage-severity prediction on datasets with reliable severity labels.
